# “Click-to-Clear”: A Strategy to Minimize Radioactivity from the Blood Pool Utilizing Staudinger Ligation

**DOI:** 10.3390/pharmaceutics15030719

**Published:** 2023-02-21

**Authors:** Nisarg Soni, Swarbhanu Sarkar, Abhinav Bhise, Yeong Su Ha, Wonchoul Park, A-Ram Yu, Virendra Kumar, Jeong Eun Lim, Young-Ran Yoon, Jeongsoo Yoo

**Affiliations:** 1 Department of Molecular Medicine, BK21 Plus KNU Biomedical Convergence Program, School of Medicine, Kyungpook National University, Daegu 41944, Republic of Korea; 2 BIOMAX. Ltd., 232, Gongneung-ro, Nowon-gu, Seoul 01811, Republic of Korea; 3 Non-Clinical Center, OSONG Medical Innovation Foundation, Cheongju 28160, Republic of Korea

**Keywords:** Staudinger ligation, click chemistry, clearing agent, tumor imaging

## Abstract

The availability of several bioorthogonal reactions that can proceed selectively and efficiently under physiologically relevant conditions has garnered the interest of biochemists and organic chemists alike. Bioorthogonal cleavage reactions represent the latest innovation in click chemistry. Here, we employed the Staudinger ligation reaction to release radioactivity from immunoconjugates, improving target-to-background ratios. In this proof-of-concept study, model systems, including the anti-HER2 antibody trastuzumab, radioisotope I-131, and a newly synthesized bifunctional phosphine, were used. Staudinger ligation occurred when biocompatible N-glycosyl azides reacted with this radiolabeled immunoconjugate, leading to cleavage of the radioactive label from the molecule. We demonstrated this click cleavage in vitro and in vivo. Biodistribution studies in tumor models showed that radioactivity was eliminated from the bloodstream, thereby improving tumor-to-blood ratios. SPECT imaging revealed that tumors could be visualized with enhanced clarity. Our simple approach represents a novel application of bioorthogonal click chemistry in the development of antibody-based theranostics.

## 1. Introduction

Since the term “bioorthogonal chemistry” first appeared in the early 2000s, there has been tremendous interest among researchers in studying life processes using a new chemical toolbox [1,2,3]. Initially, the interest revolved around “bond formation” reactions, and various new reactions were developed for applications such as cell labeling [4], protein modification [5], and antibody-based pre-targeted imaging [6,7]. Recently, a reverse approach has also been considered, and “bond cleavage” concepts are being developed [8]. Among several bioorthogonal reactions, the Staudinger ligation, azide–cyclooctyne cycloaddition, and inverse-electron-demand Diels–Alder reactions have been used for bond cleavage [9,10,11,12,13]. Researchers have found a primary use for targeted drug delivery, wherein a potent cytotoxic drug is administered as a prodrug and then activated at the site of interest via bioorthogonal click reactions.

Non-traceless copper-free Staudinger ligation is a bioorthogonal cross-linking reaction involving the initial electrophilic addition of an azide to phosphane to form an aza-ylide intermediate, which is subsequently trapped by a methoxycarbonyl group positioned near the phosphane moiety in an intramolecular fashion, resulting in the formation of an amide-linked phosphine oxide [14,15,16]. Although Staudinger ligation proceeds slowly, several in vivo experiments have demonstrated that phosphine performs better than the older generations of strained alkynes [17]. Several newer and faster bioorthogonal reactions have been reported; however, Staudinger ligation is one of the safest and most biocompatible click chemistry techniques for in vivo applications [18].

Antibodies, which are large biomolecules with an average size of approximately 150 kDa, undergo several days of slow blood clearance [19]. When used as a nuclear imaging agent, considerable time is required for their clearance from nontarget organs to achieve desirable target-to-background ratios. Because of this delay, common radioisotopes such as F-18 (*t*_1/2_ 110 min), C-11 (*t*_1/2_ 20 min), Ga-68 (*t*_1/2_ 68 min), and Cu-64 (*t*_1/2_ 12.7 h), which have short half-lives, have limited application in antibody-based imaging [20]. Long-half-life radioisotopes, such as Zr-89 (*t*_1/2_ 78.4 h), I-124 (*t*_1/2_ 100.2 h), and I-131 (*t*_1/2_ 192 h), are typically used for antibody radiolabeling to match the biological half-life of antibodies with the physical half-life of radioisotopes [21,22]. Another important application of radiopharmaceutical antibodies is the target-specific delivery of ionizing radioisotopes for therapeutic purposes [23]. However, only a small percentage of this antibody reaches the target, with the remainder circulating in the bloodstream and/or eventually being untaken by other organs such as the liver and spleen. This unnecessary radiation burden destroys healthy cells and organs, particularly the radiosensitive bone marrow [24].

The undeniable targeting ability of antibodies has led to their successful exploitation for imaging and therapeutic purposes. However, antibodies are very large biomolecules with molecular sizes of 150 kDa and blood half-life pharmacokinetics lasting several days. This results in their prolonged presence in the background, hindering their intended use. Several efforts have been made to reduce background blood uptake for targeted imaging purposes, such as the use of clearance agents and secondary antibodies. However, both of these approaches elicit immune responses and cause other safety issues [25,26]. Pre-targeted imaging strategies using bioorthogonal reporters have also been potentially useful in reducing background activity in immuno-PET imaging [27,28,29].

**Figure 1:**
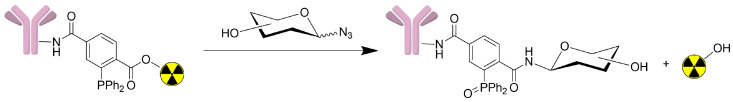
Strategy for blood pool clearance upon Staudinger ligation. The radiolabeled bioconjugate reacts chemoselectively with sugar azides to release radioactivity.

This study aimed to use Staudinger ligation click chemistry to induce selective in vivo cleavage of immunoconjugates for the release of small molecules bearing radioactivity. The liberated radioactivity can be rapidly eliminated from the bloodstream, improving the target-to-background ratio (Figure 1). Therefore, we developed a bifunctional phosphine moiety for easy radiolabeling and conjugation with antibodies. Additionally, a series of N-glycosyl azides bearing different sugar backbones and PEG linkers was prepared. The reaction between the phosphine-modified immunoconjugates and these biocompatible sugar azides was evaluated using ^1^H-NMR spectroscopy and radio-TLC. The clearance of radioactivity from the blood pool was evaluated in tumor models, and we observed an improvement in tumor-to-blood ratios.

## 2. Materials and Methods

### 2.1. General Information

All reagents and solvents used in this study were purchased from Sigma–Aldrich (St. Louis, MO, USA). Azidothymidine was purchased from ST Pharm. Co., Ltd. (Seoul, Republic of Korea). Trastuzumab antibody was purchased from Roche (Basel, Switzerland) and used without prior purification. All buffer solutions used for bioconjugation and radiolabeling were prepared using ultrapure water (>18.2 mΩ cm^−1^) obtained using the Milli-Q water purification system (Millipore, Burlington, MA, USA). TLC plates, silica gel 60 F_254_, and iTLC-SG were purchased from Merck Millipore (Darmstadt, Germany) and Agilent Technologies (Santa Clara, CA, USA). Radioiodine [^131^I]NaI was purchased from the Korea Atomic Energy Research Institute (Daejeon, Republic of Korea).

### 2.2. Synthesis and Characterization

#### 2.2.1. 1-(4-((Tert-butoxycarbonyl)oxy)phenethyl) 4-(tert-butyl) 2-(diphenylphosphaneyl) terephthalate (Compound **2**)

DMAP (0.051 g, 0.42 mmol) and *tert*-butyl (4-(2-hydroxyethyl)phenyl) carbonate (1.2 g, 5.02 mmol) were added to a stirred solution of Compound **1** (1.7 g, 4.18 mmol) in CH_2_Cl_2_ (100 mL). The solution was cooled to 0 °C, and EDC·HCl (1.20 g, 6.27 mmol) was added. The resulting solution was stirred for 10 min at 0 °C, allowed to warm to room temperature, and stirred for 2 h. The reaction was quenched with saturated aqueous NaHSO_4_ and extracted. The organic layer was washed with brine, dried over MgSO_4_, filtered, and concentrated in vacuo. The crude residue was purified by silica gel column chromatography (100% CH_2_Cl_2_) to yield Compound **2** (1.89 g, 82%) as a light yellow solid. The ^1^H NMR spectra were as follows (CDCl_3_, 500 MHz): δ 8.02 (m, 2H), 7.53 (d, 1H), 7.37–7.29 (m, 10H), 7.19 (d, 2H), 7.12 (d, 2H), 4.36 (t, 2H), 2.88 (t, 2H), 1.59 (s, 9H), and 1.45 (s, 9H).

#### 2.2.2. 3-(Diphenylphosphaneyl)-4-((4-hydroxyphenethoxy)carbonyl)benzoic Acid (Compound **3**)

Compound **2** (1.4 g, 2.23 mmol) was dissolved in 30% trifluoroacetic acid in CH_2_Cl_2_ (50 mL) and stirred for 2 h at room temperature. The solvent was removed in vacuo, and the residue was recrystallized with ether/hexane to yield a yellow solid, which was used in the next reaction without further purification (1.0 g, 95%). The ^1^H NMR spectra were as follows (CDCl_3_, 500 MHz): δ 8.09 (dd, 1H), 8.05 (dd, 1H), 7.70 (d, 1H), 7.39–7.29 (m, 9H), 7.06 (d, 2H), 6.78 (d, 2H), 4.34 (t, 2H), and 2.84 (t, 2H).

#### 2.2.3. 4-(2,5-Dioxopyrrolidin-1-yl) 1-(4-hydroxyphenethyl) 2-(diphenylphosphaneyl)terephthalate (DPTA) (Compound **4**)

N-hydroxysuccinimide (0.29 g, 2.55 mmol) and EDC·HCl (0.61 g, 3.2 mmol) were added to a stirred solution of Compound **3** (1.0 g, 2.13 mmol) in CH_2_Cl_2_ (50 mL) at room temperature. After stirring for 2 h, the solvent was concentrated and extracted using CH_2_Cl_2_/sat. NaHSO_4_. The organic layer was washed with brine, dried over MgSO_4_, filtered, and concentrated in vacuo. The crude residue was purified by silica gel column chromatography (hexane: EtOAc = 1:1) to yield Compound **4** (0.97g, 80%) as a light yellow solid. The ^1^H NMR spectra were as follows (CDCl_3_, 500 MHz): δ 8.13 (dd, 1H, *J* = 1.6 Hz), 8.11 (dd, 1H, *J* = 1.6 Hz), 8.05 (dd, *J* = 3.4 Hz), 8.03 (dd, *J* = 3.4 Hz), 7.64 (dd, 1H, *J* = 1.7 Hz), 7.35–7.24 (m, 10H), 7.02 (d, 2H, *J* = 8.4 Hz), 6.74 (d, 2H, *J* = 8.4 Hz), 4.71 (s, 1H), 4.35 (t, 2H, *J* = 7.2 Hz), 2.85 (s, 4H), and 2.80 (t, 2H, *J* = 7.2 Hz) ppm. The ^13^C NMR spectra were as follows (CDCl_3_, 125 MHz): δ 25.60, 33.89, 66.39, 115.35, 127.67, 128.70, 128.77, 129.13, 129.59, 129.74, 130.05, 130.67, 133.80, 134.01, 136.14, 136.53, 136.63, 139.84, 140.03, 142.06, 142.37, 154.24, 161.06, 165.78, 165.81, and 168.88 ppm. MS (ESI) calculated for C_32_H_26_NO_7_P^+^: *m/z* 568.1 [M+H]^+^; found: 568.3 (MH^+^).

#### 2.2.4. Synthesis of Sugar Azides

The typical reaction procedure was as follows: PEG-amine (1.1 equiv.) was added to a stirred solution of sugars (1 equivalent) in dry MeOH (10 mL). The resulting mixture was then stirred under reflux for 16 h, and TLC (silica, CH_2_Cl_2_:MeOH, 3:1) was used to evaluate the formation of a new product. The solvent was evaporated to dryness under reduced pressure. The pale yellowish gel obtained was sonicated with ethyl acetate and centrifuged. The ethyl acetate layer was then carefully decanted. The crude residue was dissolved in methanol and stirred with activated charcoal for 15 min. All compounds were isolated as colorless oil. However, upon storing for a long time, the color changed to light yellow. Freshly prepared compounds were used for biological studies, or the abovementioned charcoal decolorization step was repeated. Because sugars are heat-sensitive, all evaporation steps were performed at ≤ 40 °C. The reaction mixture turned brown during the synthesis of galactose-PEG_3_-azide. Hence, the charcoal step was repeated twice, resulting in a decreased yield.

- *Glucose-PEG_3_-azide*: D-(+)-glucose (180 mg, 1.0 mmol); 11-azido-3,6,9-trioxaundecan-1-amine (240 mg, 1.1 mmol, 1.1 equivalent); glucose-PEG_3_-azide final yield (300 mg, 79%). ^1^H NMR (D_2_O, 500 MHz): δ 2.78–2.83 (m), 2.92 (t, *J* = 5 Hz), 2.98–3.02 (m), 3.11–3.17 (m), 3.28–3.31 (m), 3.38–3.43 (m), 3.57–3.82 (m), 3.93 (d, *J* = 9 Hz), 4.55 (d, *J* = 8 Hz), and 5.14 (d, *J* = 3.5) ppm; ^13^C NMR (D_2_O, 125 MHz): δ 39.51, 50.17, 60.78, 60.94, 69.21, 69.23, 69.40, 69.55, 69.59, 69.62, 69.65, 69.91, 70.16, 71.44, 71.50, 72.77, 72.96, 74.16, 75.78, 75.95, 76.75, 76.79, 92.10, and 95.93 ppm; HRMS (FAB): *m/z* calculated for C_14_H_28_N_4_O_8_ [M+H]^+^: 381.1985; found: 381.2041.
- *Galactose-PEG_3_-azide*: D-(+)-galactose (500 mg, 1.0 mmol); 11-azido-3,6,9-trioxaundecan-1-amine (666 mg, 1.1 mmol, 1.1 equivalent); galactose-PEG_3_-azide final yield (308 mg, 29%). ^1^H NMR (D_2_O, 500 MHz): δ 3.09 (t, *J* = 5 Hz), 2.98–3.02 (m), 3.38–3.44 (m), 3.54–3.57 (m), 3.61(d, *J* = 4Hz), 3.64–3.68 (m), 3.74 (d, *J* = 15 Hz), 3.83 (d, *J* = 5 Hz), 3.84 (d, *J* = 5 Hz), 3.9–4.01 (m), 4.49 (d, *J* = 8 Hz), and 5.17 (d, *J* = 3.5 Hz) ppm; ^13^C NMR (D_2_O, 125 MHz): δ 39.20, 50.14, 60.97, 61.17, 68.34, 68.74, 69.16, 69.21, 69.30, 69.45, 69.49, 69.54, 69.56, 69.63, 70.46, 71.87, 72.79, 75.14, 92.27, and 96.44 ppm; HRMS (FAB): *m/z* calculated for C_14_H_28_N_4_O_8_ [M+H]^+^: 381.1985; found: 381.1983.
- *Lactose-PEG_3_-azide*: α-lactose (1 g, 1.0 mmol); 11-azido-3,6,9-trioxaundecan-1-amine (0.7 g, 1.1 mmol, 1.1 equivalent.); lactose-PEG_3_-azide final yield (1.1 g, 70%). ^1^H NMR (D_2_O, 400 MHz): δ 2.73–2.79 (m), 2.92–2.98 (m), 3.10–3.15 (m), 3.37–3.49 (m), 3.51–3.84 (m), 3.92 (d, *J* = 8 Hz), 4.32 (d, *J* = 8 Hz), 4.53 (d, *J* = 8 Hz), and 5.09 (d, *J* = 3.6 Hz) ppm; ^13^C NMR (D_2_O, 100 MHz): δ 39.90, 44.55, 50.49, 61.39, 68.90, 69.58, 69.73, 69.76, 69.86, 69.88, 69.93, 70.00, 70.45, 70.48, 71.31, 72.87, 72.97, 74.17, 74.72, 75.14, 75.70, 75.93, 78.64, 78.98, 89.87, 92.17, 96.12, and 103.26 ppm; HRMS (FAB): *m/z* calculated for C_20_H_38_N_4_O_13_ [M+H]^+^: 543.2514; found: 543.2514.
- *Maltose-PEG_3_-azide*: D-(+)-maltose (350 mg, 1.0 mmol); 11-azido-3,6,9-trioxaundecan-1-amine (245.5 mg, 1.1 mmol, 1.1 equivalent); maltose-PEG_3_-azide final yield (450 mg, 81%). ^1^H NMR (D_2_O, 400 MHz): δ 2.74–2.77 (m), 2.91–2.97 (m), 3.08–3.15 (m), 3.28 (t, *J* = 9.2 Hz), 3.36–3.76 (m), 3.91 (d, *J* = 8 Hz), 4.51 (d, *J* = 8 Hz), 5.08–5.09 (m), and 5.27 (s) ppm; ^13^C NMR (D_2_O, 100 MHz): δ 39.84, 44.52, 50.48, 60.81, 69.58, 69.73, 69.88, 69.93, 70.00, 70.28, 70.48, 71.63, 72.00, 72.09, 73.01, 73.18, 73.21, 73.58, 74.34, 74.89, 75.67, 76.56, 77.00, 77.22, 77.40, 77.56, 89.86, 92.23, 93.12, and 99.90 ppm; HRMS (FAB): *m/z* calculated for C_20_H_38_N_4_O_13_ [M+H]^+^: 543.2514; found: 543.2518.
- *Maltose-PEG_6_-azide*: D-(+)-maltose (100 mg, 1.0 mmol); O-(2-Aminoethyl)-O′-(2-azidoethyl) pentaethylene glycol (112.6 mg, 1.1 mmol, 1.1 equivalent); maltose-PEG_6_-azide final yield (170 mg, 86%). ^1^H NMR (D_2_O, 400 MHz): δ 2.84–2.88 (m), 3.09–3.12 (m), 3.17 (t, *J* = 7.2 Hz), 3.28–3.45 (m), 3.48 (dd, *J* = 7.6, 4.8 Hz), 3.53 (t, *J* = 7.2 Hz), 3.67–3.68 (m), 3.69–3.87 (m), 3.91 (d, *J* = 7.2 Hz), and 5.18 (d, *J* = 3.2 Hz) ppm; ^13^C NMR (D_2_O, 125 MHz): δ 39.20, 44.19, 50.16, 60.51, 60.60, 60.74, 60.90, 69.21, 69.23, 69.34, 69.36, 69.47, 69.52, 69.55, 69.58, 69.63, 69.96, 70.15, 71.30, 71.68, 71.72, 71.78, 72.67, 72.69, 72.82, 72.86, 72.89, 73.22, 74.01, 74.57, 75.35, 76.30, 76.81, 77.05, 77.20, 77.23, 89.55, 91.89, 95.78, and 99.62 ppm; HRMS (FAB): *m/z* calculated for C_26_H_50_N_4_O_16_ [M+H]^+^: 675.3300; found: 675.3298.
- *Maltose-PEG_11_-azide*: D-(+)-maltose (100 mg, 1.0 mmol); O-(2-Aminoethyl)-O′-[2-azidoethyl] decaethylene glycol (140.9 mg, 1.1 mmol, 1.1 equivalent); maltose-PEG_11_-azide final yield (132 mg, 51%). ^1^H NMR (D_2_O, 400 MHz): δ 2.84–2.88 (m), 3.09–3.18 (m), 3.28–3.33 (m), 3.41 (t, *J* = 4 Hz), 3.48 (dd, *J* = 7.6, 3.2 Hz), 3.53 (t, *J* = 7.2 Hz), 3.62–3.69 (m), 3.82 (t, *J* = 4 Hz), 3.91 (d, *J* = 7.2 Hz), and 5.18 (d, *J* = 3.2 Hz) ppm; ^13^C NMR (D_2_O, 125 MHz): δ 39.23, 44.23, 50.18, 60.54, 60.61, 60.76, 60.91, 69.38, 69.61, 69.99, 70.14, 71.32, 71.71, 71.75, 71.81, 72.69, 72.83, 72.89, 72.92, 73.23, 74.02, 75.37, 76.22, 76.89, 77.20, 77.31, 89.58, 91.91, 95.81, and 99.67 ppm; HRMS (FAB): *m/z* calculated for C_36_H_70_N_4_O_21_ [M+H]^+^: 895.4611; found: 895.4608.

#### 2.2.5. Synthesis of 4-(2-Hydroxyethyl)-2-iodophenol

Under an atmosphere of air, 30 mg of 2-(4-hydoxyphenyl) ethanol (1.0 equivalent) and THF (4 mL) were added to a dry flask (10 mL) fitted with a magnetic stirrer. These were stirred with the dissolved starting material. Then, N-iodosuccinimide, 54 mg (1.1 equivalent) was added and stirred for another 2 h at room temperature (27 °C). The desired 4-(2-hydroxyethyl)-2-iodophenol (2.9 mg, 5.1 %) was isolated using HPLC (X-Bridge C18 column, 150 mm × 4.6 × 5 µm) as a light yellow solid. The desired product was eluted for around 20 min using the following gradient method: 0–20% B in 5 min, 20–90% B in 25 min, and 90–0% B in 40 min. Mobile phase H_2_O (A) and CH_3_CN (B) both contained 0.1% trifluoroacetic acid. ^1^H NMR (CDCl_3_, 500 MHz) δ 2.79 (t, *J*–*6.5*): 7.56, 3.84 (t, *J*–*6.5*), 6.96 (d, *J*–*8*), 7.13 (dd, *J_1_*–*2, J_2_*–*8*), and 7.56 (d, *J*–*2*) ppm; ^13^C NMR (CDCl_3_, 125 MHz) δ:37.70, 65.57, 115.03, 130.86, 132.74, 138.39, 153.50, and 161.43 ppm; HRMS (FAB) *m/z* calculated for C_8_H_9_IO_2_ [M]^+^: 263.9647, found 263.9650 (Figures S27–S30).

### 2.3. Radiolabeling and Bioconjugation

A diphenyl phosphine terephthalate analog (DPTA) was radiolabeled with ^131^I by modifying the standard chloramine-T radioiodination method [30]. Briefly, DPTA (10 µL, 0.6 mg/mL ethanol) was added to a vial containing [^131^I]NaI (37 MBq) in 100 µL of phosphate-buffered saline (PBS). Chloramine-T (10 µL, 5 mg/mL) was added and incubated for 3 min. The reaction was terminated by adding sodium metabisulfite (15 µL, 12 mg/mL). The labeling yield was monitored using radio-TLC (iTLC-SG; saline). For conjugation with trastuzumab, 2.5 mg of antibody in borate buffer (0.1 M, pH 9.4, 200 µL) was added to the radioiodinated DPTA and incubated for 2 h at 37 °C. The reaction progress was monitored using radio-TLC (silica; methanol). The radiolabeled bioconjugate was purified by passing it through a PD-10 column to collect 200-µL fractions and then concentrated using YM-10 centrifugal filters.

Radiolabeling of 2-(4-hydroxyphenyl)ethanol was performed by mixing (10 µL, 1 mg/mL ethanol) 2-(4-hydroxyphenyl) ethanol in PBS (100 µL) and [^131^I]NaI (13 MBq). Chloramine-T (10 µL, 5 mg/mL) was added to the mixture and incubated for 5 min at 25 °C. The reaction was quenched with sodium metabisulphite (5 µL, 12 mg/mL) and monitored using radio-TLC (silica; ethyl acetate:methanol [95:5]). Radiolabeled 4-(2-hydroxyethyl)-2-[^131^I] iodophenol (**10**) was purified using reverse-phased HPLC equipped with a WATERS X-Bridge C18 column (150 mm × 4.6 × 5 µm) and eluted with methanol:water (55:45) at a flow rate of 1 mL/min. Collected fractions were evaporated to dryness, reconstituted in PBS, and used for further studies. To confirm the radiolabeled product, standard nonradioactive 4-(2-hydroxyethyl)-2-iodophenol was injected for detection at 220 nm, while the radiolabeled product was detected using a radiodetector (Bioscan Flow-Count detector, Berlin, Germany).

### 2.4. In Vitro Stability Studies

Approximately 50 µL (1.85 MBq) of radiolabeled bioconjugate and 450 µL of PBS or fetal bovine serum (FBS) were mixed and incubated at 37℃ with moderate agitation. Aliquots were collected starting at 30 min and up to 7 h after mixing, run on silica-TLC using methanol as the mobile phase, and scanned using a radio-TLC scanner.

### 2.5. In Vitro Staudinger Ligation

[^131^I]DPTA-trastuzumab (1.85 MBq) was mixed with N-glycosyl azide (50 mg) at 37 °C. Aliquots were collected at 30 min and 1, 2, 4, 6, 8, 12, and 24 h after mixing, and TLCs were checked using iTLC-SG and saline.

### 2.6. Effect of Solvent on In Vitro Staudinger Ligation

A total of 50 µL of radiolabeled DPTA (1.85 MBq) and 50 mg maltose-PEG_3_-azide were mixed, and the volume was adjusted to 500 µL using a solvent. The resulting mixture was incubated at 37 °C for 7 h. The cleavage was examined via radio-TLC analysis using a silica/CH_2_Cl_2_: MeOH (95:5) TLC system.

### 2.7. Cell Viability Assay

In vitro cytotoxicity was evaluated using the 3-(4,5-dimethylthiazol-2-yl)-2,5-diphenyltetrazolium bromide (MTT) assay [31]. Human embryonic kidney HEK293 and HER2-overexpressing murine NIH3T6.7 cells (1.0 × 10^4^ cells/well) were seeded in a 96-microwell plate at 37 °C overnight and incubated with 10, 100, or 1000 μg DPTA for 7 and 24 h at 37 °C under 5% CO_2_. MTT (10 μL; 5 mg/mL in PBS) was added to each well and incubated for an additional 3 h. Formazan crystals were dissolved in 100 μL DMSO for 30 min. Optical density was measured at 550 nm using a microplate reader (Molecular Devices). The yellow tetrazolium salt of MTT was reduced to purple formazan crystals in metabolically active cells. Viability was determined based on the ratio of the optical density in the treated cells to that in the untreated control cells. The results are presented as mean ± standard deviation (n = 3).

### 2.8. Animal Studies

All animal experiments were performed in accordance with the Animal Care and Use Committee requirements of Kyungpook National University (approval no. 2019-0101, approval date 19 July 2019). All animals were housed at proper temperature and humidity in a 12-h day/12-h night cycle with adequate water and standard feed. For SPECT imaging and biodistribution studies, female BALB/c nude mice models were used (20–22 g, 6 weeks old). Xenograft tumor models were prepared by inoculating 5 × 10^6^ NIH3T6.7 cells into the right flank of the mice. The NIH3T6.7 tumor cell line was maintained in high-glucose Dulbecco’s modified Eagle’s medium supplemented with 10% FBS and 1% penicillin–streptomycin. Cells were incubated at 37 °C under 5% CO_2_ and maintained until they attained 80% confluence. Cancer cells were harvested with 0.25% trypsin–EDTA to develop tumor models.

### 2.9. Ex Vivo Analysis of Staudinger Ligation in Blood

[^131^I]DPTA-trastuzumab (1.85–2.5 MBq, 200-μL PBS) was administered to normal BALB/c mice (male, 19–20 g, 7 weeks old) intravenously. Mice were divided into two groups (n = 3 each): control and azide treatment. Maltose-PEG_3_-azide (30 mg in 200-μL normal saline) was administered intravenously at 1 h and 3 h post-injection and blood (200 μL) was withdrawn at 5 h post-injection. Whole blood was centrifuged (10,000 rpm, 4 °C, 10 min) to separate serum, which was spotted on iTLC to develop in saline. In the remaining azide-treated serum (~20 µL), 5 µL of 4-(2-hydroxyethyl)-2-[^131^I]iodophenol (**10**) was mixed and spotted on iTLC to develop in saline.

### 2.10. Biodistribution Studies

Six-week-old ICR male mice were purchased from Hyochang Bio and used for subsequent biodistribution studies. Under anesthesia with 1–2% isoflurane in O_2_, the purified radiobioconjugate [^131^I]DPTA-trastuzumab (0.74 MBq, 200 μL saline) was injected via the tail vein. N-glycosyl azides (30 mg in 200 μL saline) were injected at 1 h, 1 h & 5 h, and 1 h & 3 h and 5 h, respectively, post-injection of the [^131^I]DPTA-trastuzumab. Seven hours after injecting [^131^I]DPTA-trastuzumab, the mice (n = 3) were euthanized under anesthesia (isoflurane). Blood and various tissues of interest were immediately removed, and the blood was wiped using cotton gauze, weighed, and counted on a γ counter (Wallach, Turku, Finland). For the control studies, only [^131^I]DPTA-trastuzumab (0.74 MBq, 200 μL saline) was injected via the tail vein. No N-glycosyl azides were injected. As another control study, 4-(2-hydroxyethyl)-2-[^131^I]iodophenol (**10**) (0.74 MBq, 200 μL saline) was injected into the tail vein of ICR male mice, following which biodistribution studies were conducted at 30 min and 1, 4, and 24 h post-injection. The percentage of the injected dose was calculated by comparison with a diluted standard solution derived from the injected solution. Data are reported as the percentage of injected dose per gram (%ID/g) of wet tissue.

### 2.11. SPECT Imaging

SPECT/CT imaging was performed using a four-head nanoSPECT/CT scanner (Mediso, Budapest, Hungary). The animals were anesthetized with isoflurane (1–2% in O_2_), placed in the prone position, restrained with adhesive tape, and scanned. SPECT scans were obtained with four-headed multiplexing multipinhole collimator. Each head was fitted with an application-specific tungsten collimator with 16 pinholes. For this study, we used a mice aperture (M3 High energy Mouse Whole Body aperture), which comprises a total of 64 individual 1.7 mm diameter pinholes. The axial FOV is extended using a step-and-shoot helical scan defining a range of mouse whole body. The energy peak for the camera was set at 364.5 keV and energy window was 328–400 keV (±10%). For imaging studies, radiolabeled [^131^I]DPTA-trastuzumab (3.7–5.5 MBq each) was injected into the tail vein of nude mice bearing NIH3T6.7 xenografts. After the indicated times, SPECT images were obtained using a nanoSPECT/CT scanner. SPECT images were reconstructed using the manufacturer’s software (InterView Fusion ver3.0) at an isotropic voxel output size of 234 μm. CT and SPECT scans were coregistered.

### 2.12. Statistical Analysis

Statistically significant values were determined as comparisons between two groups and multiple groups, conducted by paired or unpaired Student’s *t* test (two-tailed), or two-tailed analysis of variance. The experimental data were presented as mean ± SD. * *p* < 0.05, ** *p* < 0.01, *** *p* < 0.001. *p* Value < 0.05 was considered as statistically significant.

## 3. Results

### 3.1. Synthesis of Prosthetic Group DPTA and Characterization

**Scheme 1:**
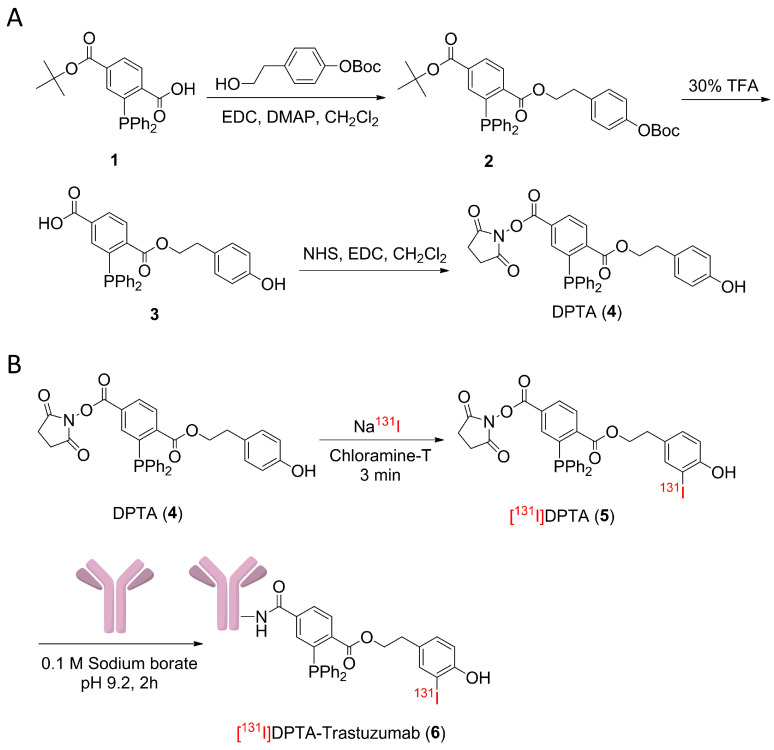
Synthesis of DPTA (**A**) and its radiolabeling and bioconjugation with trastuzumab antibody (**B**).

As a model compound for evaluating our hypothesis, a DPTA (**4**) was designed. The target molecule was synthesized from terephthalate Derivative **1** in three steps [32] (Scheme 1A). The carboxylic group underwent esterification using Boc-protected primary alcohol to generate Compound **2**, followed by TFA-mediated deprotection to generate Compound **3**. Finally, the free acid in **3** was activated with N-hydroxysuccinimide to generate the final compound, DPTA (**4**). DPTA was characterized by ^1^H- and ^13^C-NMR and high-resolution mass spectrometry (Figures S1–S3).

### 3.2. Synthesis of Sugar Azides

**Figure 2:**
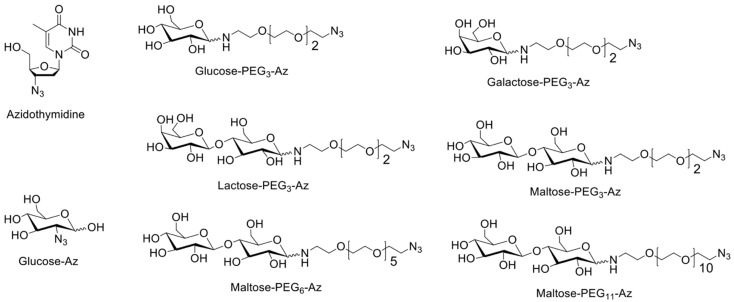
All azide compounds were used for Staudinger ligation screening studies.

Sodium azide, the most basic form of azide, is widely used as a bactericidal agent in mammalian cell cultures; due to extreme toxicity, it cannot be directly used for any in vivo study [33]. Other forms of water-insoluble azides are rendered unusable for in vivo applications. Previous reports have used azide-modified metabolic precursors of sugars to visualize glycans on the cell surface [14]. This inspired us to develop several N-glycosyl azides for this study (Figure 2). Starting from commercially available low-cost monosaccharides and disaccharides, the desired glycosyl azides were obtained simply by refluxing both precursors in anhydrous methanol for 16 h (Figures S4–S22). During the reaction, the solution turned pale yellow. Excess PEG-amine was removed using ethyl acetate, and decolorization was carried out using activated charcoal. The newly synthesized N-glycosyl-PEG_n_-azide was nontoxic and deemed safe for use in further biological experiments (Figures S23 and S24).

### 3.3. ^1^H-NMR Kinetic Study

**Figure 3:**
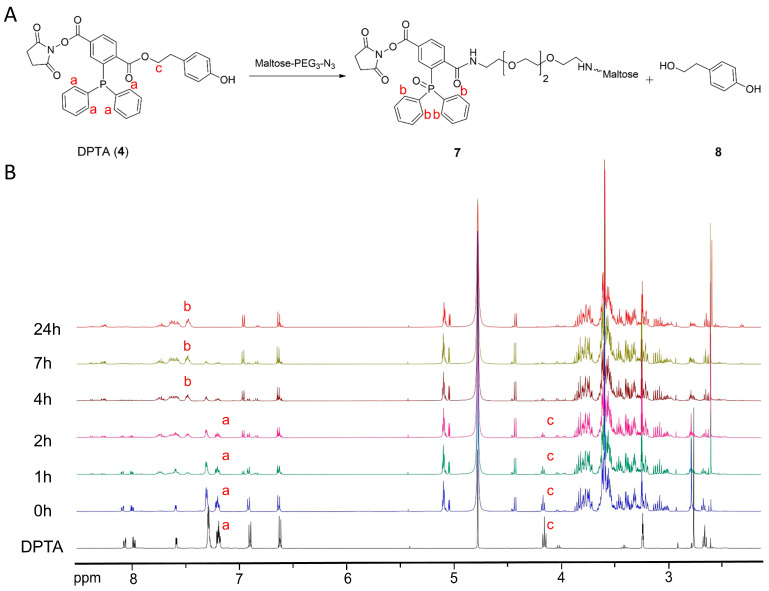
The reaction between DPTA and maltose-PEG_3_-azide leading to the generation of phosphine oxide (7) and the elimination of (8) (**A**). ^1^H-NMR spectroscopic kinetic analysis of the reaction between DPTA and maltose-PEG_3_-azide (**B**). Marks “a”, “b”, and “c” in Figure 3B are the protons described in Figure 3A.

Subsequently, ^1^H NMR spectrometry was applied to monitor the reaction between DPTA (**4**) and sugar azide in dry [D_4_] MeOH at 25 °C. An NMR kinetics study was performed by mixing two equivalents of maltose-PEG_3_-azide with one equivalent of DPTA (**4**) (Figure 3).

The reaction between DPTA (**4**) and the sugar azide generates intermediate (**7**), where sugar gets attached to DPTA via an amide bond and the phosphine group is converted to phosphine oxide. The reaction also generated free 4-hydroxyphenylethyl alcohol (**8**) (Figure 3A). Thus, a steady change in the chemical shift in the surrounding environment of phosphorous was hypothesized. Simultaneously, a steady alteration in the peak intensity of methylene proton next to the cleavable ester bond was also hypothesized. In Figure 3B, four equivalent aromatic protons (‘a’ in Figure 3B) adjacent to the phosphorous appear as a multiplet at 7.18–7.21 ppm, whereas the triplet at 4.16 ppm corresponds to the methylene proton (‘c’ in Figure 3B) adjacent to the ester oxygen.

As revealed by the NMR kinetics experiment, most DPTA (**4**) was converted to phosphine oxide (**7**) within 1 h of the reaction. The intensity of the triplet at 4.16 ppm immediately started to decrease because of the ester bond cleavage in DPTA. The peak intensity decreased rapidly and completely disappeared within 4 h of the reaction. The reaction was monitored for up to 24 h. However, no further considerable changes in peak intensity were observed, confirming the completion of the reaction within 4 h. Apart from the decrease in the peak intensity at 4.16 ppm, a decrease in peak intensity at 7.18–7.21 ppm was also noticed with a simultaneous increase at 7.45 ppm (‘b’ in Figure 3B).

### 3.4. Radioiodination and Bioconjugation

DPTA was designed such that it enabled radioiodination at one end and conjugation with biomolecules at the other end. It was radioiodinated at the tyrosine end with a slight modification to the standard chloramine-T method. After confirming the reactivity between the cold phosphine analog and the sugar azide, we radiolabeled DPTA (**4**) with ^131^I. The radiolabeling yield of DPTA with ^131^I was approximately 95% (Figure S25). Radioiodinated DPTA was used without purification for further biomolecule conjugation. The NHS group, present at the other end of the phosphine compound, facilitated the easy attachment of biomolecules. The NHS group of DPTA was attached to the amine group of the antibody (trastuzumab) by its reaction in the presence of borate buffer for 2 h (Scheme 1B). The reaction progress was monitored using radio-TLC, and the final conjugation yield was about 50%. This radiolabeled bioconjugate was purified using a PD-10 column and concentrated using YM-10 centrifugal filters.

### 3.5. Synthesis and Radiolabeling of 4-(2-Hydroxyethyl)-2-iodophenol

In one step, 4-(2-hydroxyethyl)-2-iodophenol was synthesized from 2-(4-hydroxyphenol) ethanol (Figure S27). Due to the aromatic electrophilic substitution at the highly reactive orthoposition, mono and di-iodo-substituted compounds were formed as the major and minor products, respectively. The desired product was purified via HPLC. Mono-iodinated 4-(2-hydroxyethyl)-2-iodophenol and di-iodinated 4-(2-hydroxyethyl)-2,6-diiodophenol, were eluted at 4.7 and 9.9 min, respectively (Figure S31D,E). The radiochemical yield of 4-(2-hydroxyethyl)-2-[^131^I]iodophenol (**10**) was 99% (Figure S31B). Two peaks were observed in the HPLC analysis of the radiolabeled mixture. The major peak at 5.1 min corresponded to the desired product 4-(2-hydroxyethyl)-2-[^131^I]iodophenol (**10**), and a minor peak at 10.2 min corresponded to the di-iodinated side product (Figure S31F). Only 4-(2-hydroxyethyl)-2-[^131^I]iodophenol (**10**) was isolated for the following uses.

### 3.6. In Vitro Stability

**Figure 4:**
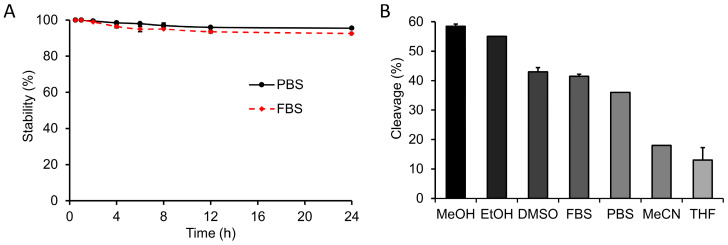
In vitro stability of [^131^I]-DPTA-trastuzumab in PBS and FBS up to 24 h (n = 2) (**A**). Solvent effect on Staudinger ligation (n = 2) (**B**).

For Staudinger ligation to take place, the radiolabeled DPTA-trastuzumab moiety must remain intact under biological conditions. To determine the stability, the radiolabeled immunoconjugate was incubated in PBS and FBS at 37 °C for 24 h. It was deduced that over 95% and about 92% of the immunoconjugates remained intact in PBS and FBS, respectively (Figure 4A). This indicated that it can be safely used in subsequent in vivo experiments.

### 3.7. Effect of Solvent on In Vitro Staudinger Ligation

The TLC studies conducted with radiolabeled DPTA and maltose-PEG_3_-azide revealed that the highest cleavage occurred in MeOH (59%), followed by EtOH (55%), DMSO (43%), and PBS (36%; Figure 4B). Acetonitrile and tetrahydrofuran (THF) gave the least cleavages of 18% and 13%, respectively. This variable cleavage, depending on the solvent used, can be attributed to the variable interactions between the two reacting partners. Radiolabeled DPTA and Mal-PEG_3_-azide were completely soluble in MeOH or ethanol, whereas maltose-PEG_3_-azide was sparingly soluble in DMSO, thereby decreasing the homogeneity of the reaction medium. Although maltose-PEG_3_-azide was completely soluble in FBS and PBS, radiolabeled DPTA was found to be less soluble in nature. The poor yield in the case of acetonitrile and THF was attributed to the poor solubility of both components in those solvents.

### 3.8. Cleavage of Sugar Azides

**Figure 5:**
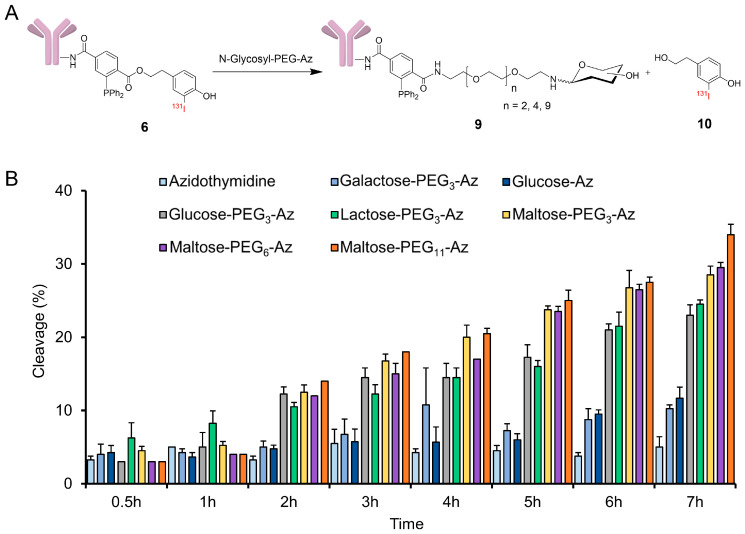
The reaction between [^131^I]DPTA-trastuzumab (**6**) and N-glycosyl azides led to the elimination of radioactivity (**A**). Graph representing the cleavage observed at the corresponding time points (n = 4) (**B**).

The reaction between radiolabeled DPTA and N-glycosyl azides was observed using the radio-TLC method. On the iTLC plate with saline as the mobile phase, the radiolabeled DPTA remained at the origin, whereas the cleaved product (**10**) moved to the front (Figure 5A). This was the basis of the cleavage screening studies. The cleavage percentage was calculated from the integration ratio of the peak at the base (**6**) and front peaks (**10**) in the radio-TLC chromatograms. The time-dependent reactivity of the radiolabeled immunoconjugates toward various sugar azides was monitored (Figure 5B). First, commercially available non-PEGylated sugar azides, azidothymidine, and glucose azides were used. These compounds did not show any remarkable cleavage at up to 5 h of the reaction. The cleavage by glucose azide increased to 11.7% ± 1.5% over 7 h, and the cleavage caused by azidothymidine remained at 5% ± 1.41% over the same time. It can be argued that the lack of PEG linkers makes it difficult for azide groups to react with antibody-conjugated phosphine.

Next, the newly synthesized PEGylated N-glycosyl azide was tested. Even at early time points (at 1 h), the cleavage performances of the PEGylated sugar azides were better than those of the non-PEGylated sugar azides, with a maximum of 8.3% ± 1.7% for lactose-PEG_3_-azide. The cleavage pattern maltose-PEG_3_-azide was found to be very promising; it increased from 12.5% ± 1.0% to 28.5% ± 1.2% within 2 to 7 h (Figure 5B). The effect of the PEG linker length on the cleavage rates was also examined. The non-PEGylated glucose azide exhibited much less cleavage (11.7% ± 1.5%) as compared to the PEGylated glucose azide analog (23.0% ± 1.4%) at 7 h. Additionally, maltose was modified to have two more different linker arms (PEG_6_ and PEG_11_). Maltose-PEG_11_-azide showed the best cleavage of 34.0% ± 1.4%, followed up by that of maltose-PEG_6_-azide with a cleavage of 29.5% ± 0.7%. This suggests that having long linker arms helps the N-glycosyl azides to react effectively with the phosphine analog.

### 3.9. Blood Analysis of In Vivo Staudinger Ligation

To determine the amount of the cleaved compound (**10**) in the blood (Figure 5A), the serum of both control and azide-treated mice by radio-TLC was analyzed. The radiolabeled antibody remained at the origin, whereas radiolabeled 4-(2-hydroxyethyl)-2-[^131^I]iodophenol (**10**) moved toward the TLC front. As expected, only the intact radiolabeled antibody was found at the origin in the control mouse serum (non-azide-treated), while in the serum of azide-treated mice, around 6% of total activity moved with an R_f_ value of 0.56. The R_f_ is the ratio between the solute distance traveled and the solvent distance traveled on TLC plates. When purified 4-(2-hydroxyethyl)-2-[^131^I]iodophenol (**10**) was mixed with the azide-treated serum and spotted on a TLC plate again, the peak at R_f_ value of 0.56 increased to 63% from 6%, indicating that the peak at R_f_ 0.56 is the released compound (**10**) (Figure S33). Overall, this study demonstrates that Staudinger ligation proceeds in vivo, and radioactivity can be removed from radiolabeled antibodies by treating azide compounds.

### 3.10. Biodistribution Studies

**Figure 6:**
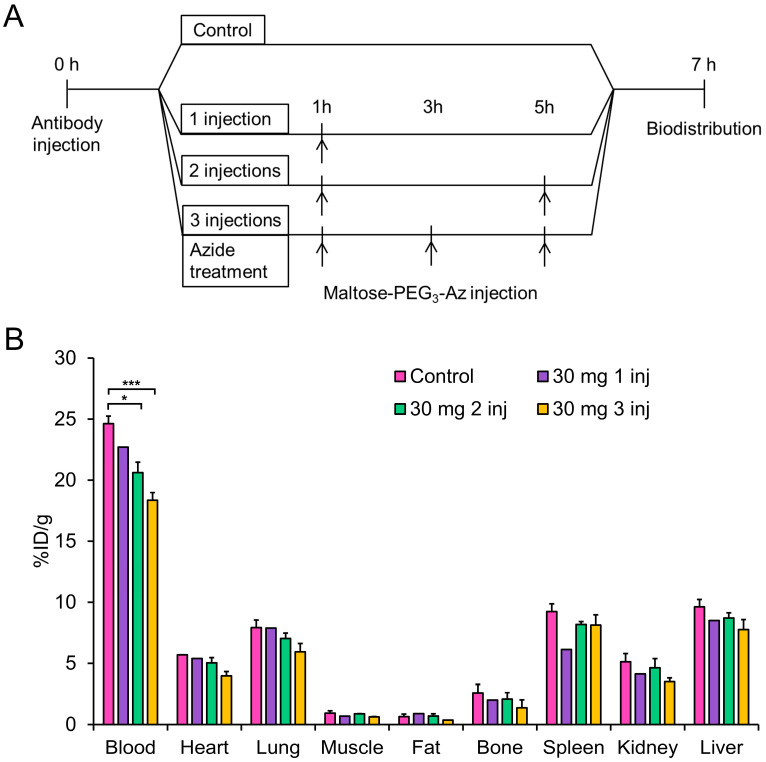
A schematic representation demonstrating the injection of radiolabeled antibody followed by maltose-PEG_3_-azide and finally biodistribution (**A**). Biodistribution data of ICR mice after injection of [^131^I]DPTA-trastuzumab followed by changing the number of maltose-PEG_3_-azide injections (**B**) (n = 2). * *p* < 0.05 and *** *p* < 0.001.

In this study, the suitability of Staudinger ligation-based elimination under in vivo conditions was tested. Radioiodinated DPTA was conjugated with trastuzumab and injected into the ICR mice. After allowing the radiolabeled bioconjugate to circulate for an hour, N-glycosyl azide (maltose-PEG_3_-azide) was injected. The first screening was to determine the number and intervals of the injections. The mice were divided into four groups: the first group received only one dose of sugar azide (30 mg per mouse) 1 h after the antibody injection; the control group did not receive any sugar azide injection. The second group received two doses of sugar azide with a time difference of 4 h between them, whereas the third group received three doses of sugar azide every 2 h (Figure 6A). The results indicated that the third group gave the most promising blood clearance among the lots (Figure 6B). Multiple sugar azide injections resulted in a higher chance of interaction with the phosphine-antibody conjugate circulating in the blood pool, leading to better clearance. When the injection amount of maltose-PEG_3_-azide was reduced from 30 mg to 20 mg and 10 mg, the blood clearance was gradually decreased (Figure S26). The activity retention in blood was 18.3 ± 0.6, 20.1 ± 1.0, and 21.5 ± 0.3 ID/g for 30, 20, and 10 mg, respectively (Figure S26B). Overall, the dose optimization study suggests that 30 mg of sugar azide injected thrice reduces blood pool activity effectively.

**Figure 7:**
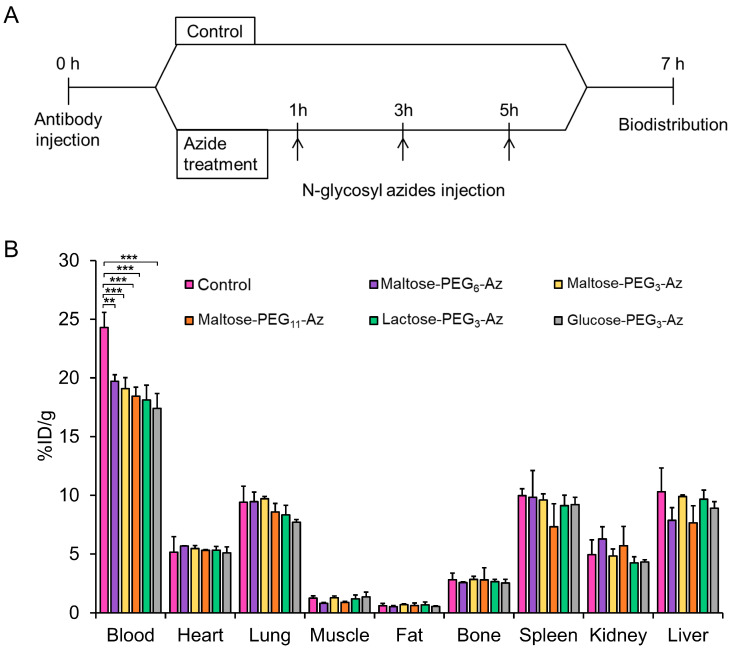
A schematic representation depicting the injection of radiolabeled antibody followed by sugar-azides (**A**). Biodistribution data of ICR mice demonstrating the blood pool radioactivity elimination upon reaction with the respective N-glycosyl azides (n = 3) (**B**). ** *p* < 0.01 and *** *p* < 0.001.

Finally, the in vivo cleavage rates of various N-glycosyl azides based on glucose, lactose, and maltose with different PEG sizes were compared (Figure 7). All sugar azides exhibited similar reactivity with the phosphine-conjugated antibody, leading to an approximately 25% faster clearance of blood activity. More specifically, glucose-PEG_3_-azide showed the best clearance from the blood (17.4 ± 1.2%ID/g) as compared to other sugar azides (18.1 ± 1.3%ID/g and 19.1% ± 1.0%ID/g for lactose-PEG_3_-azide and maltose-PEG_3_-azide, respectively; Figure 7B). Additionally, the effect of the PEG linker length was studied. It was theorized that increasing the PEG length would enhance the blood pool retention of the sugar azides and thus enhance the radioisotope clearance upon Staudinger ligation. However, it was observed that the blood clearance of maltose-PEG_11_-azide was only marginally better than that of maltose-PEG_3_-azide (18.5 ± 0.8%ID/g vs. 19.1 ± 1.0%ID/g, respectively). Contrastingly, an improved blood pool clearance was not observed by increasing the PEG linker length. This may be attributed to the fact that the increased linker size is still not large enough to increase blood retention significantly.

Moreover, another biodistribution study of 4-(2-hydroxyethyl)-2-[^131^I]iodophenol (**10**) was performed in normal mice to substantiate our hypothesis that the background activity clearance, especially in the blood, can be accelerated by selectively cleaving the radioactivity moiety from antibody conjugates. In the blood analysis study of in vivo Staudinger ligation (Figure S32), it was confirmed that the side product (**10**) was released from the antibody conjugated by Staudinger ligation upon injecting azides. The small organic molecule of 4-(2-hydroxyethyl)-2-[^131^I]iodophenol (**10**), as expected, showed extremely fast body excretion. Within 30 min post-injection of (**10**), most of the activity was cleared out from the body. Only 1.12% ± 0.29% and 4.46% ± 0.57% of injected doses per gram were found in the blood and kidney, respectively, which was dramatically lower than the >20%ID/g value of the radiolabeled antibody conjugated in the blood even at 7 h postinjection of [^131^I]DPTA-trastuzumab (**6**). These data indicate that the cleaved radioactivity gets eliminated quickly from the body via renal excretion.

### 3.11. SPECT Imaging and Biodistribution Studies in Tumor Models

After proving that the Staudinger ligation indeed occurred in vivo and could eliminate radioactivity from the blood pool, it was decided to apply the same strategy to tumor models to increase tumor-to-background ratios. HER2-positive NIH3T6.7 tumor models were prepared and injected with radiolabeled DPTA-antibody bioconjugate, followed by a series of sugar azide injections as described previously (Figure 7A).

**Figure 8:**
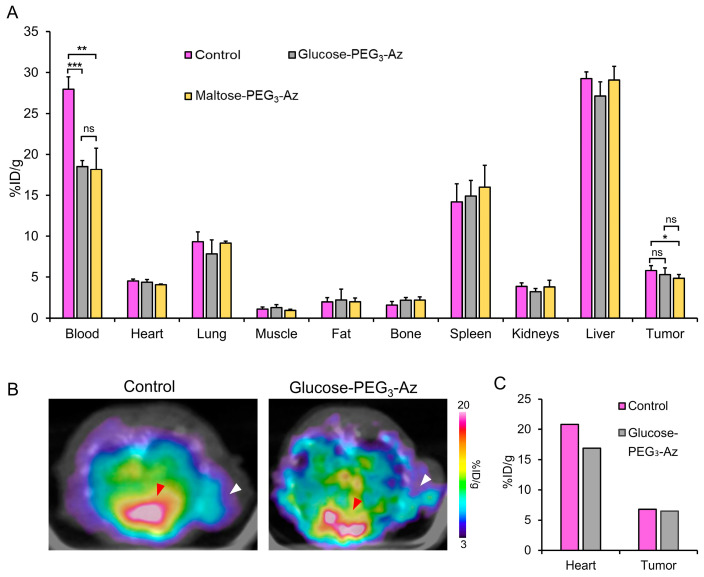
SPECT and Biodistribution study in female nude mice bearing NIH3T6.7 tumors at 7 h postinjection of [^131^I] -DPTA-trastuzumab. Biodistribution (**A**). SPECT images (n = 1 each); Control and glucose-PEG_3_-azide (**B**), and quantification data of glucose-PEG_3_-azide (**C**). The red arrowhead indicates the heart, whereas the white arrowhead indicates tumor. ns represents not statistically significant. * *p* < 0.05, ** *p* < 0.01, and *** *p* < 0.001.

The biodistribution pattern in NIH3T6.7 tumor models was very similar to that of the normal ICR mice (Figure 7B). The radioactivity uptake in most organs, including tumors, was comparable between the control group and N-glycosyl azide-treated groups (Figure 8A). Only blood showed significantly different uptakes; 28.0 ± 1.2%ID/g, 18.5 ± 0.7%ID/g, and 18.2 ± 2.6%ID/g for the control, glucose-PEG_3_-azide, and maltose-PEG_3_-azide groups, respectively. The N-glycosyl azide-treated groups showed 34–35% lower blood uptakes compared with the control group. However, the tumor uptake in the sugar azide-treated groups decreased only by 8.9–16.5%, leading to a 22–28% increase in the tumor-to-blood (T/Bl) ratios compared with the control group (T/Bl 0.21 ± 0.03, 0.29 ± 0.05, and 0.27 ± 0.05 for the control, glucose-PEG_3_-azide and maltose-PEG_3_-azide groups, respectively).

Finally, an animal SPECT imaging study was performed. At 1 h post-injection of [^131^I]DPTA-trastuzumab, three serial injections of glucose-PEG_3_-azide were followed, then imaged at 7 h (Figure 8B). In parallel with the biodistribution study, the blood activity signal in the heart of the control mouse was more intense compared with that in the glucose-PEG_3_-azide-treated mouse. Although tumor uptake signals were comparable between the two mice, tumor signals were more clearly recognized from the surrounding background in the sugar azide-treated mouse because of lower background noises. The glucose-PEG_3_-azide-treated mouse showed 15.4% lower activity in the heart compared to that of the control mouse. The tumor uptakes were very comparable (5.6%ID/g and 5.3%ID/g for the glucose-PEG_3_-azide-treated and control mice, respectively; Figure 8C).

## 4. Discussion

This study reports a novel cleavable radiotracer that can be rapidly cleared from the blood pool to improve tumor-to-blood ratios. Owing to their slow pharmacokinetics, the radiolabeled antibodies remain in circulation for a longer period [24]. This causes a radiation burden and makes an accurate diagnosis difficult. Bioorthogonal reactions have been previously developed to improve tumor-to-blood ratios [1]. Among the pool of bioorthogonal reactions, phosphine-azide-based Staudinger ligation has been shown to be safe in vivo. Since its introduction a century ago [34], Staudinger ligation has been primarily explored using radiolabeled antibodies, such as that employed in the bond formation pre-targeted approach developed by Vugts et al. [35]. In the present study, the opposite bond cleavage strategy with Staudinger ligation chemistry was employed, which substantially improved ligation under in vivo conditions while maintaining tumor-bound activity. Therefore, the new phosphine prosthetic group DPTA was reacted with various sugar azides (Figure 1). Biocompatible and commercially available sugars, including glucose, maltose, galactose, and lactose, were azide-functionalized with different PEG lengths (Figure 2). Azide toxicity in HEK293 and NIH3T6.7 cells was determined to be safe for biological use (Figures S23 and S24). Subsequently, the release of the radioisotope from the radioimmunoconjugate was systematically studied using various ex vivo and in vivo experiments. First, a ^1^H-NMR kinetic study of maltose-PEG_3_-azide revealed that 4 h of incubation was sufficient for the complete cleavage of the DPTA tracer, releasing the 4-hydroxyphenylethyl alcohol (Figure 3). After confirming cleavage, the DPTA tracer was radioiodinated with the oxidant chloramine-T and conjugated with the HER2+ trastuzumab antibody (Scheme 1B). On examining the in vitro stability of the radioimmunoconjugate, it was found to be up to 95% and 92% stable in PBS and FBS, respectively (Figure 4A).

Although we demonstrated the cleavage of DPTA using NMR, we substantiated the adequate time required for the effective cleavage of the radioimmunoconjugate using radio-TLC. Therefore, the cleavage of PEGylated and non-PEGylated sugar azides was compared. It was found that the gradual increase in PEG length from 3, 6, and 11 units improved cleavage with time compared with non-PEGylated azides (Figure 5). After confirming the cleavage efficiency and biocompatibility, we initiated the ultimate goal of this study. A series of biodistribution studies were performed on normal mice to identify the optimal time gap between injections and the quantity of sugar azides required. A 30-mg injection showed a 25.6% improvement in blood clearance compared with the control, whereas the 10- and 20-mg injections showed 12.6% and 18.3% improvement in blood clearance, respectively (Figure S26). Overall, 30 mg of the sugar azides with a time gap of 2 h in three injections for a total of 90 mg demonstrated the best in vivo cleavage of the radioimmunoconjugate with rapid clearance and decrease in radioactivity in the blood pool (Figure 6 and Figure 7).

The SPECT and biodistribution studies (Figure 8) of the cleavable radioimmunoconjugate in the HER2+ NIH3T6.7 tumor xenografts revealed a 35% improvement in blood clearance, while that in other normal organs remained comparable. Although tumor uptake was moderate at an earlier time point, no effect was observed on tumor-bound activity after treatment with azides, as observed in the blood. This phenomenon correlates with that observed in previous studies; at earlier time points, the tumor uptake remains low, and the clearing agent should not hamper tumor uptake [36,37]. Overall, the primary goal of this study was successfully achieved, and our findings could be useful for improving the clinical application of bulkier antibody-based tumor-imaging agents.

## 5. Conclusions

This proof-of-concept study demonstrates the novel use of Staudinger ligation click chemistry as a tool to eliminate radioactivity and clear the background for vivid tumor visualization. Newly synthesized DPTA can easily conjugate with an antibody. In addition, it can effectively eliminate radioactivity upon clicking on appropriate N-glycosyl azides. Various bioorthogonal click reactions have been employed for many applications related to chemical biology. However, this is the first time that Staudinger ligation-based click chemistry has been used to develop radioactivity clearance agents. The clearance strategy used here will eventually be useful in several clinical situations, ranging from antibody-based diagnosis to therapeutic prognosis monitoring. This novel application of click chemistry will serve as an important contribution to the ever-growing bioorthogonal chemistry toolbox.

## Data Availability

Additional supporting data can be found in Supplementary Materials (Figures S1–S33).

## Supplementary Materials

The following supporting information can be downloaded at https://www.mdpi.com/article/10.3390/pharmaceutics15030719/s1, Figures S1–S3: Characterization of DPTA (**4**) tracer; Figure S4: Synthesis of N-glycosyl azides; Figures S5-S22: Characterization of N-glycosyl azides; Figure S23: Cell viability of N-glycosyl azides in NIH3T6.7 cells; Figure S24: Cell viability of N-glycosyl azides in HEK293 cells; Figure S25: Radio-TLC chromatograms of radiotracer and bioconjugates; Figure S26: Dose optimization study by injecting different amounts of N-glycosyl azides; Figures S27–S30: Synthesis of 4-(2-hydroxyethyl)-2-iodophenol and spectroscopic characterization; Figure S31: Radiolabeling of 4-(2-hydroxyethyl)-2-[^131^I]iodophenol and HPLC analyses; Figure S32: Radio-TLC analysis of in vivo Staudinger ligation in mice; Figure S33: Biodistribution of 4-(2-hydroxyethyl)-2-[^131^I]iodophenol in normal ICR mice.
