# Supplementary material for: “Click-to-Clear”: A Strategy to Minimize Radioactivity from the Blood Pool Utilizing Staudinger Ligation
Source: Pharmaceutics. 2023 Feb 21;15(3):719. doi: 10.3390/pharmaceutics15030719 (PMC10057653; doi:10.3390/pharmaceutics15030719)
Supplement: Supplementary file 1 [file pharmaceutics-15-00719-s001.zip › pharmaceutics-2117277-supplementary.pdf]

pharmaceutics

Supporting Information

Contents:

Figures S1–S3. Characterization of DPTA (**4**) tracer

Figure S4. Synthesis of N-glycosyl azides

Figures S5–22. Characterization of N-glycosyl azides

Figure S23. Cell Viability of N-glycosyl azides in NIH3T6.7 cells

Figure S24. Cell Viability of N-glycosyl azides in HEK293 cells

Figure S25. Radio thin-layer chromatograms of radiotracer and bioconjugates

Figure S26. Dose optimization study by injecting different amount of N-glycosyl azides

Figures S27–30: Synthesis of 4-(2-hydroxyethyl)-2-iodophenol and spectroscopic characterization

Figure S31: Radiolabeling of 4-(2-hydroxyethyl)-2-[^{131}I]iodophenol and HPLC analyses

Figure S32: Radio-TLC analysis of in vivo Staudinger ligation in mice

Figure S33: Biodistribution of 4-(2-hydroxyethyl)-2-[^{131}I]iodophenol in normal ICR mice

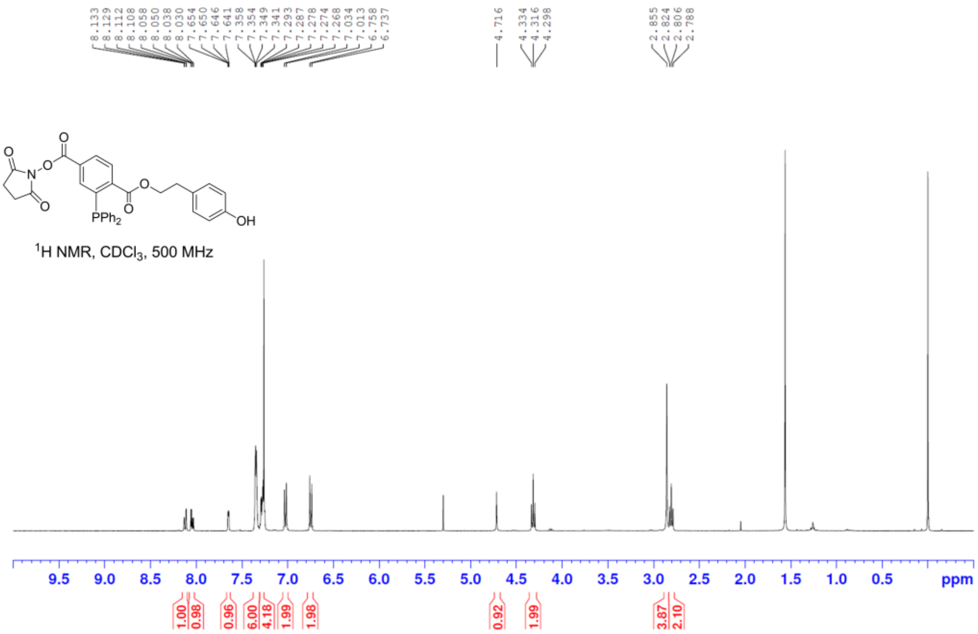

Figure S1. ^{1}H-NMR spectra for tracer DPTA (**4**).

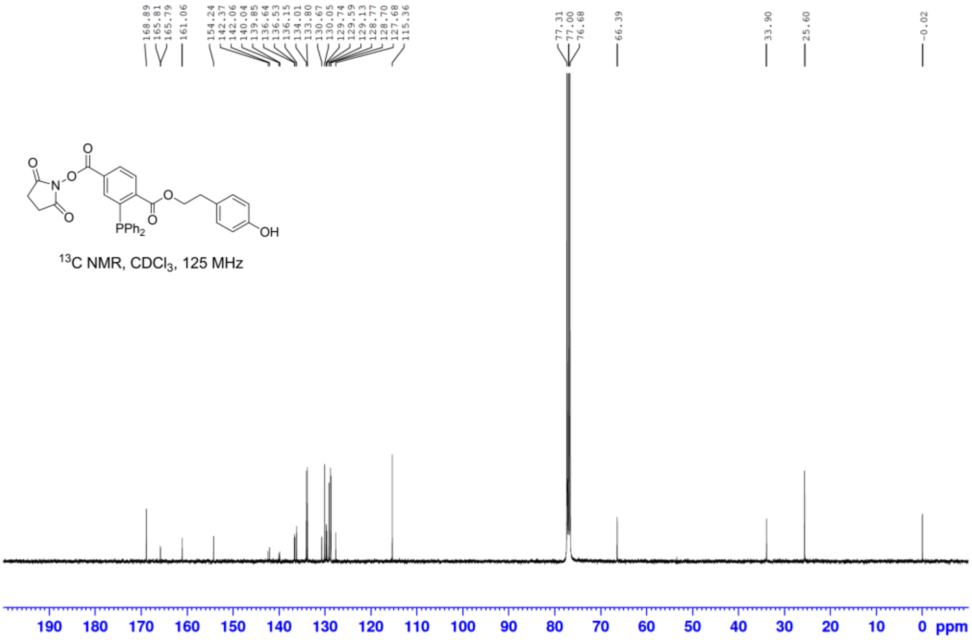

Figure S2. ^{13}C-NMR spectra for tracer DPTA (**4**).

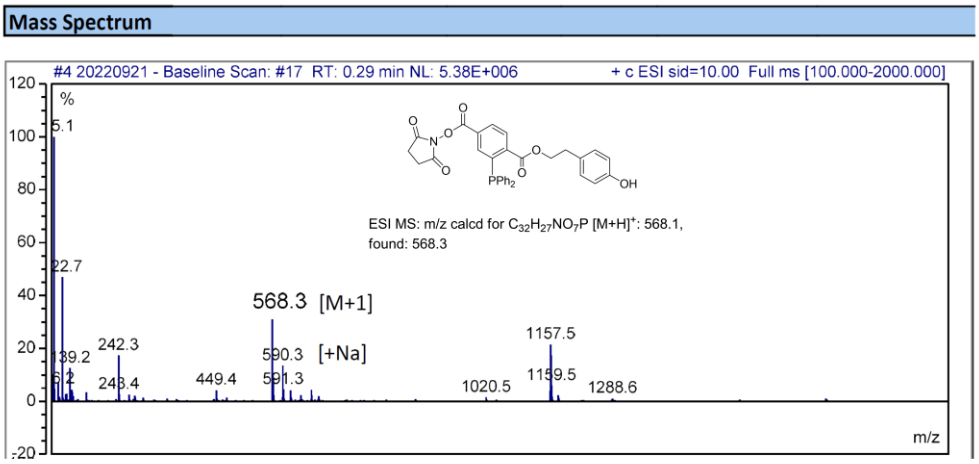

Figure S3. ESI-mass spectrometric data for tracer DPTA (**4**).

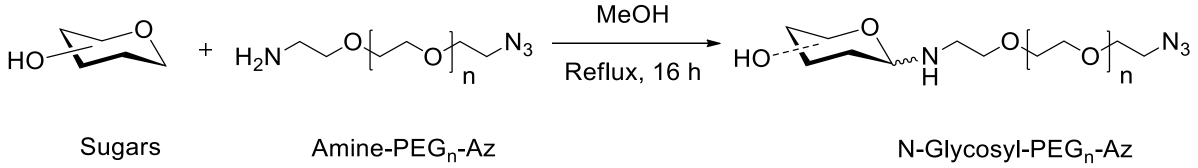

Figure S4. Schematic representation for the synthesis of N-glycosyl azides.

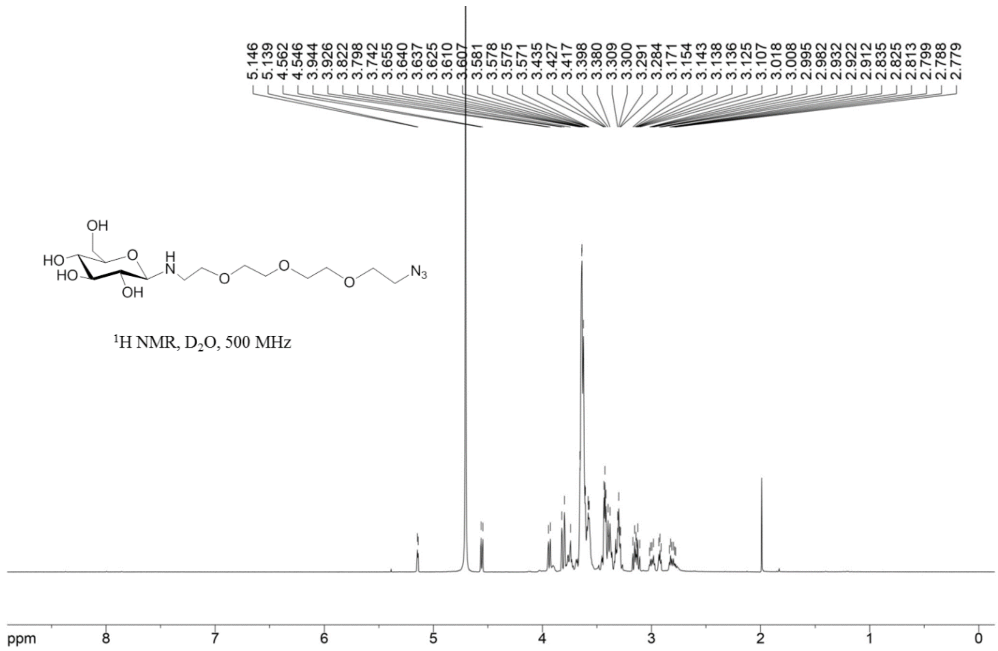

Figure S5. ^{1}H-NMR spectra for glucose-PEG$_3$-azide.

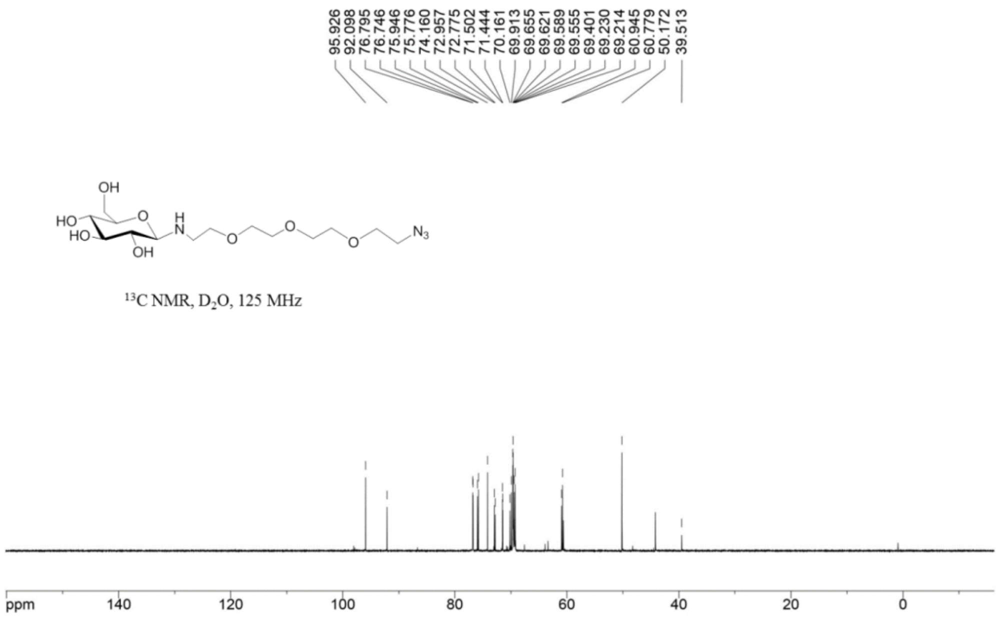

Figure S6. ^{13}C-NMR spectra for glucose-PEG$_3$-azide.

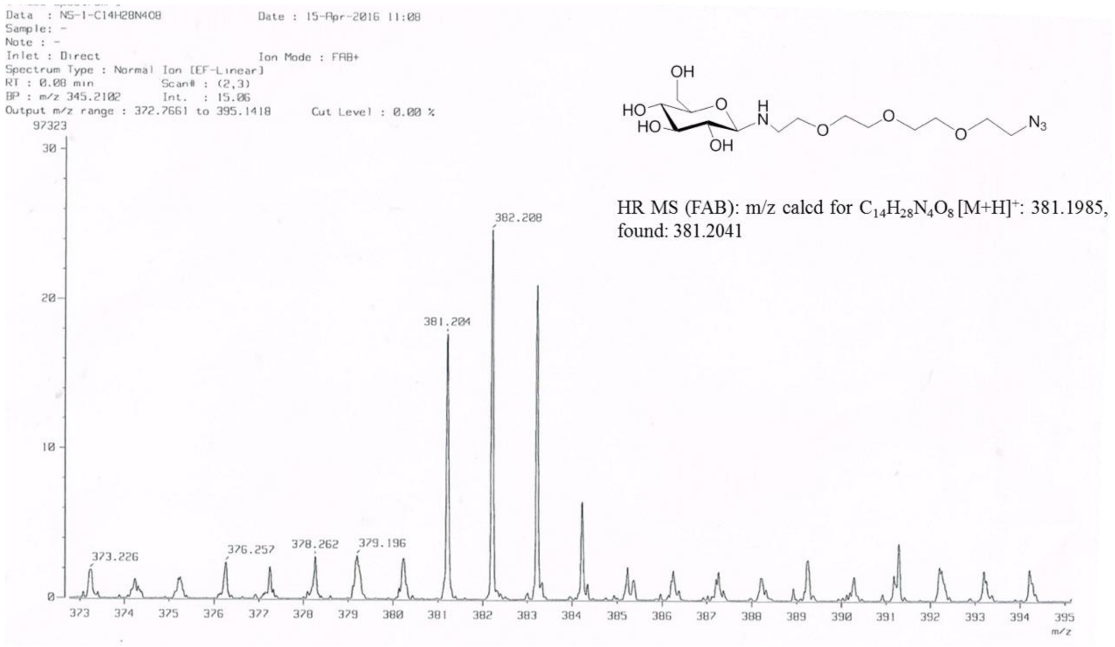

Figure S7. High resolution mass spectroscopic data for glucose-PEG$_3$-azide.

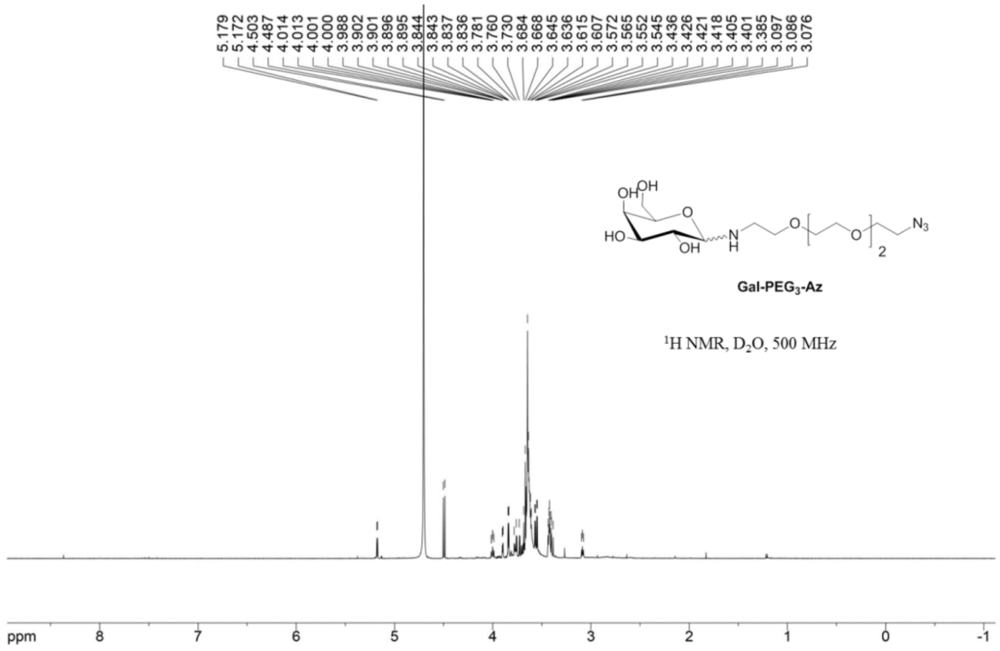

Figure S8. ^{1}H-NMR spectra for galactose-PEG$_3$-azide.

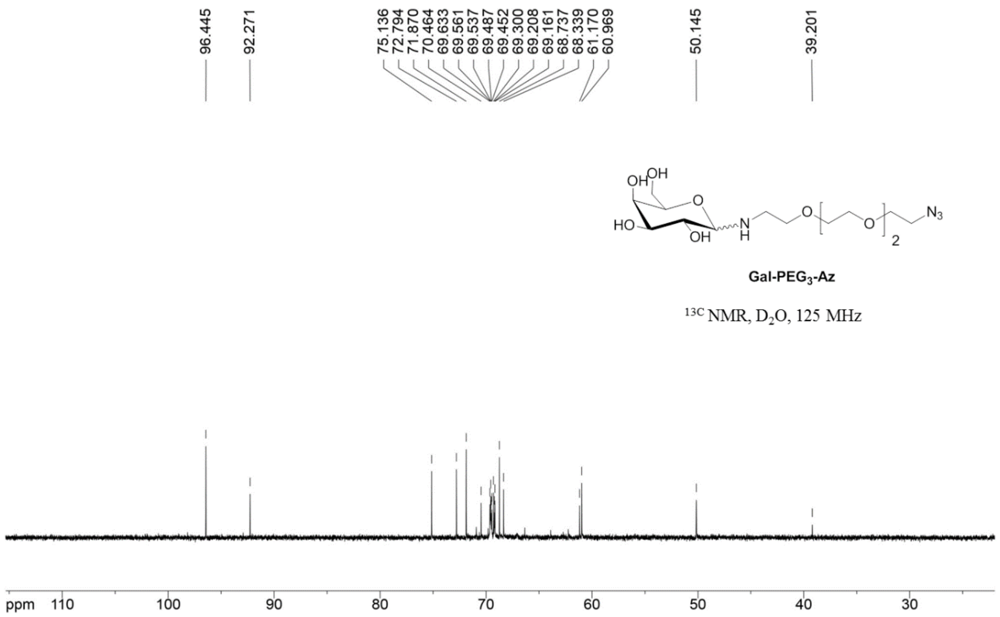

Figure S9. ^{13}C-NMR spectra for galactose-PEG$_3$-azide.

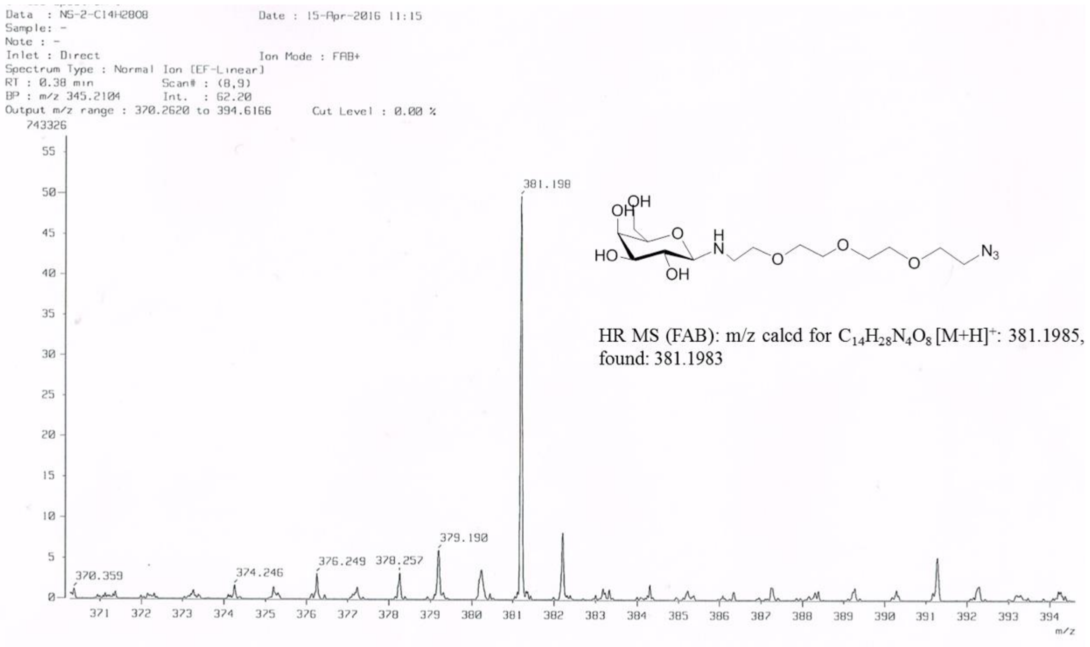

Figure S10. High resolution mass spectroscopic data for galactose-PEG3-azide.

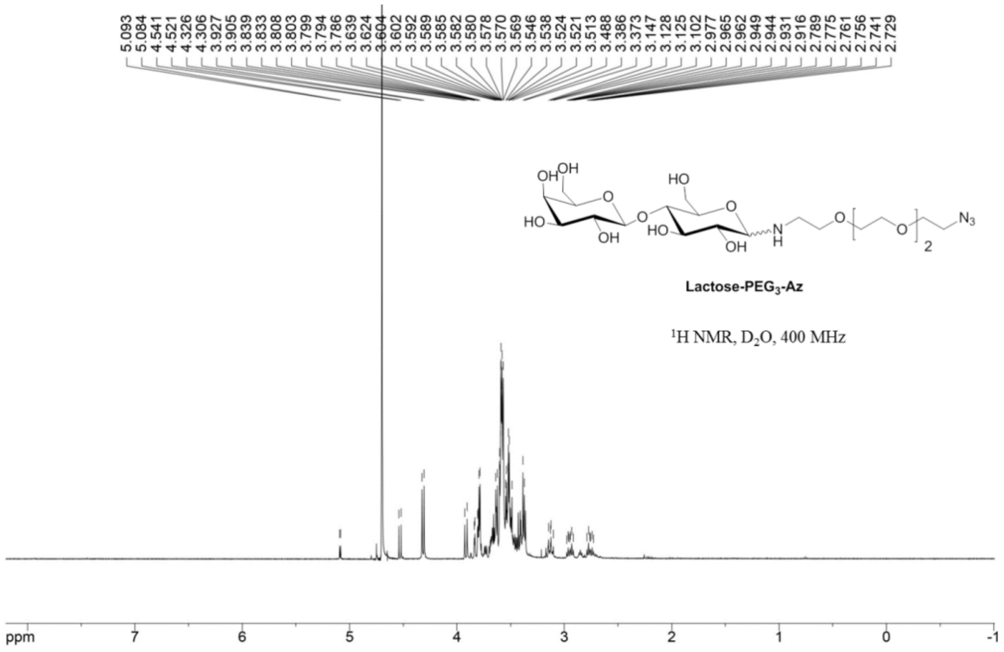

Figure S11. ^{1}H-NMR spectra for lactose-PEG3-azide.

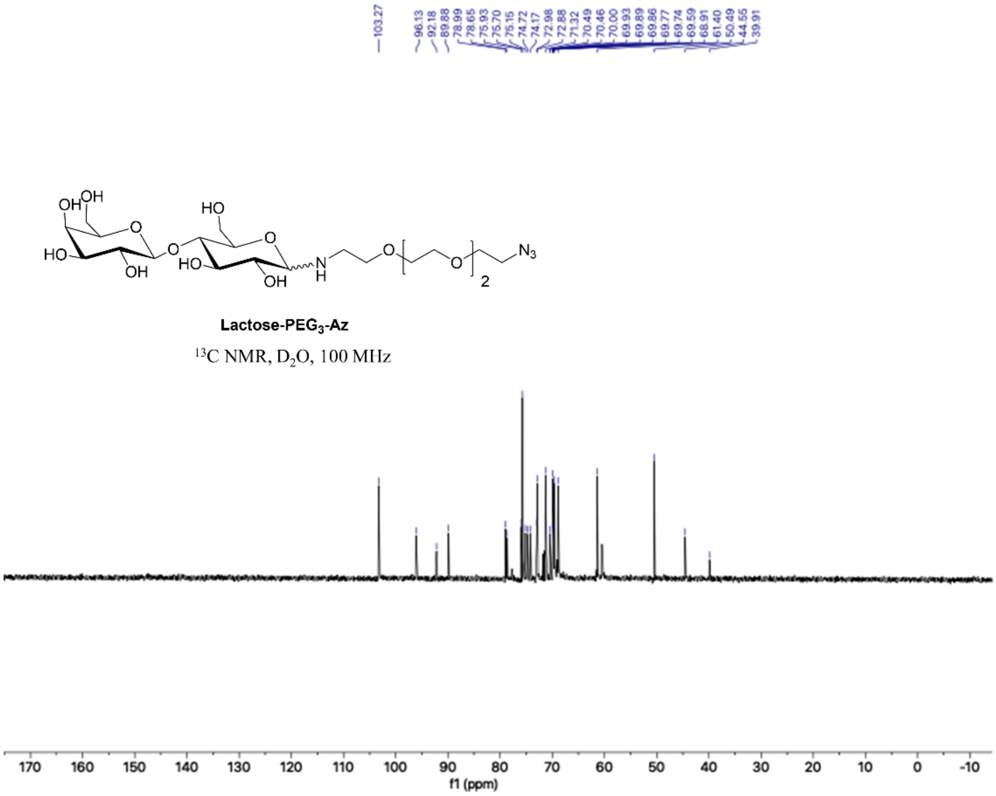

Figure S12. ^{13}C-NMR spectra for lactose-PEG$_3$-azide.

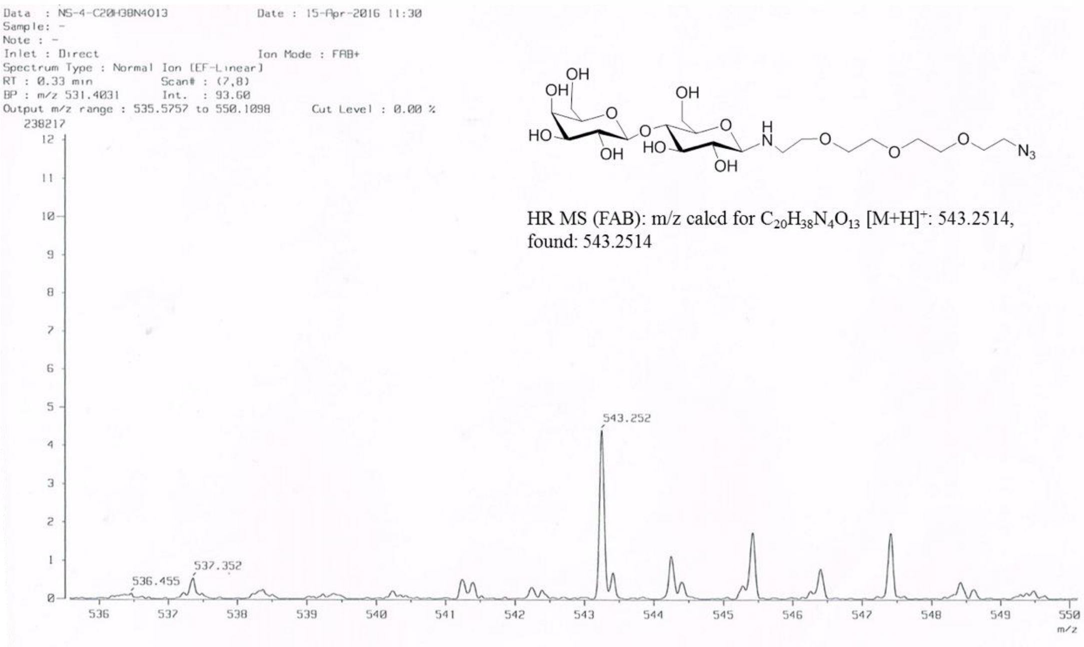

Figure S13. High resolution mass spectroscopic data for lactose-PEG$_3$-azide.

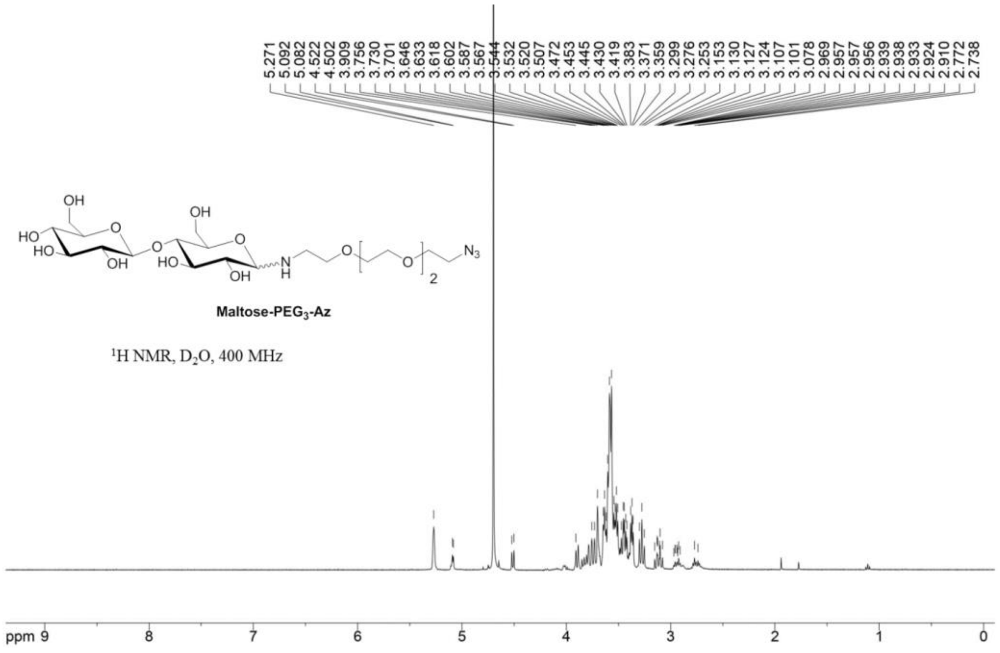

Figure S14. ^{1}H-NMR spectra for maltose-PEG$_3$-azide.

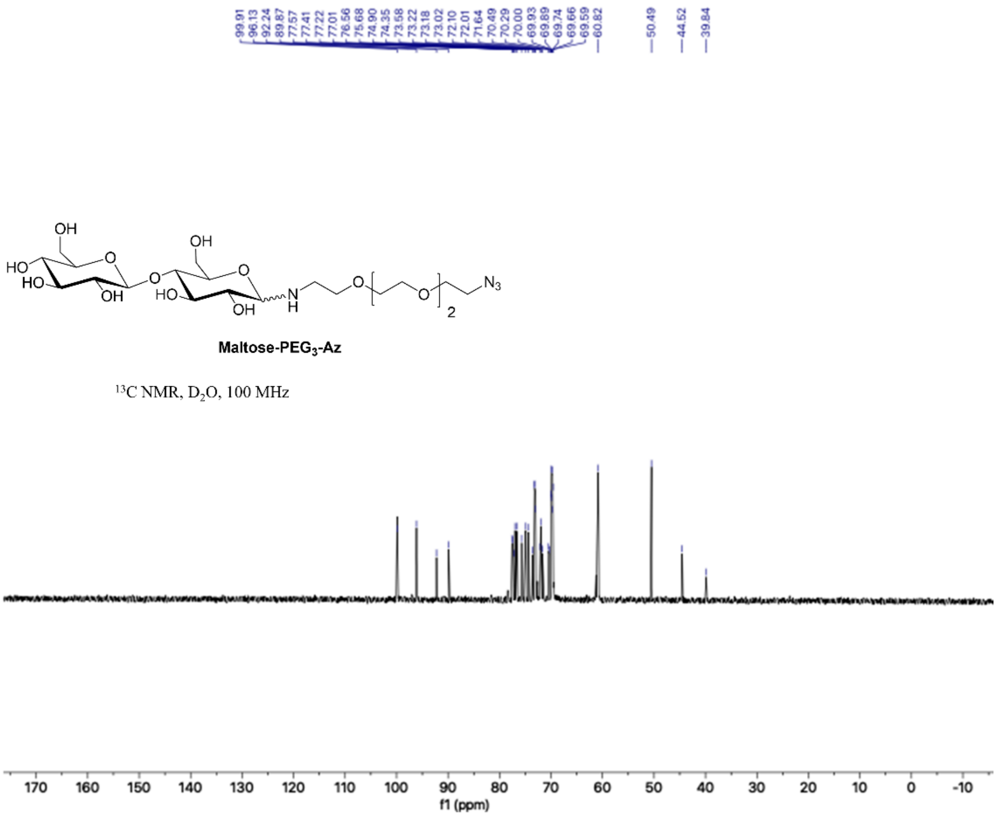

Figure S15. ^{13}C-NMR spectra for maltose-PEG$_3$-azide.

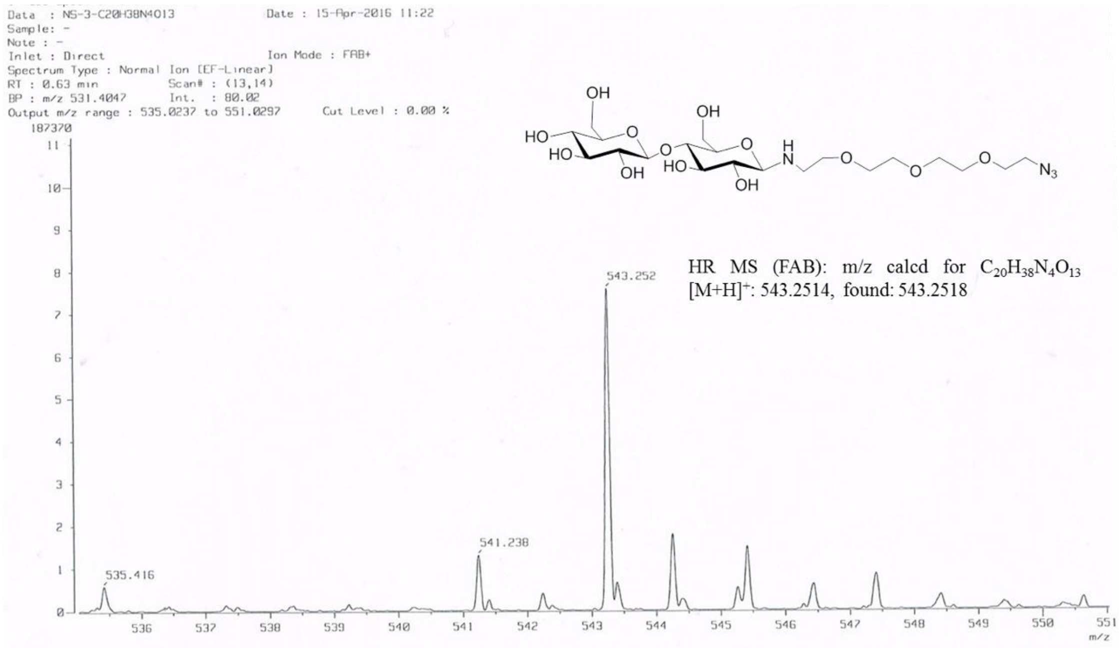

Figure S16. High resolution mass spectroscopic data for maltose-PEG3-azide.

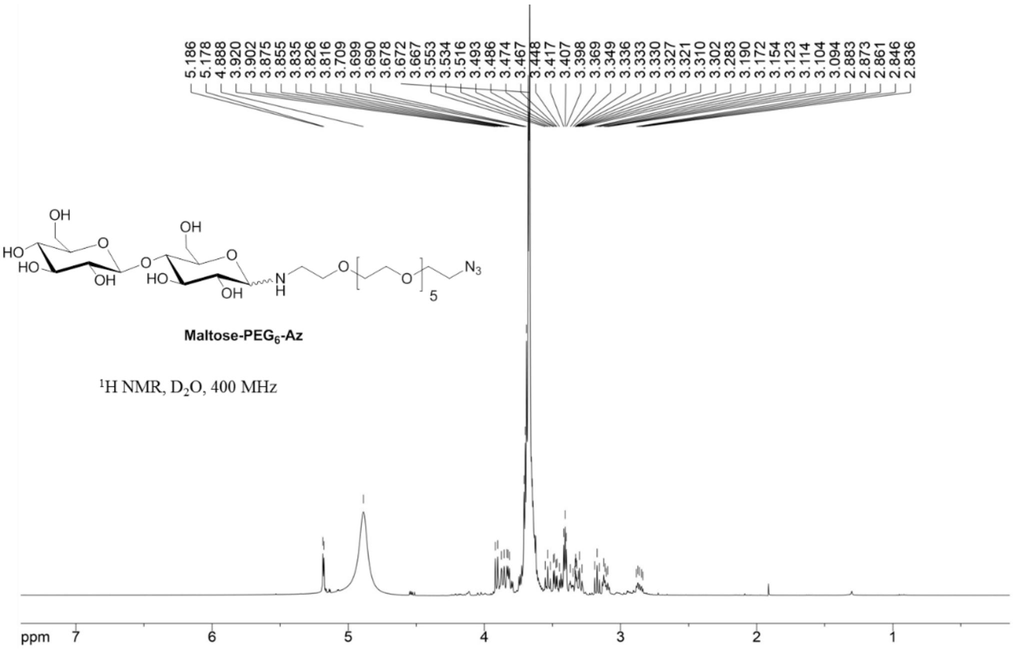

Figure S17. 1H NMR spectra for maltose-PEG6-azide.

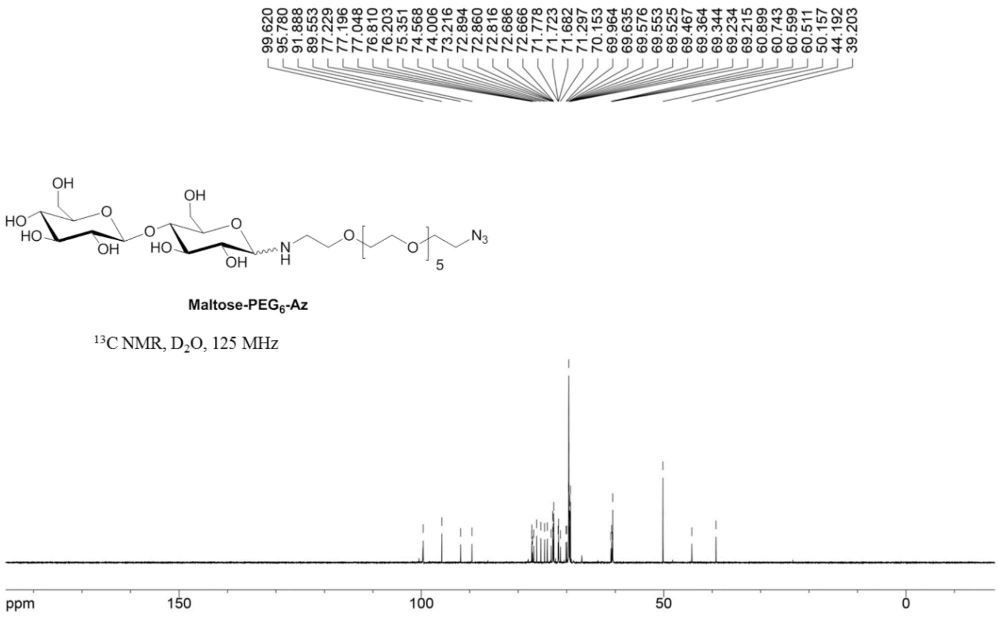

Figure S18. ^{13}C NMR spectra for maltose-PEG$_6$-azide.

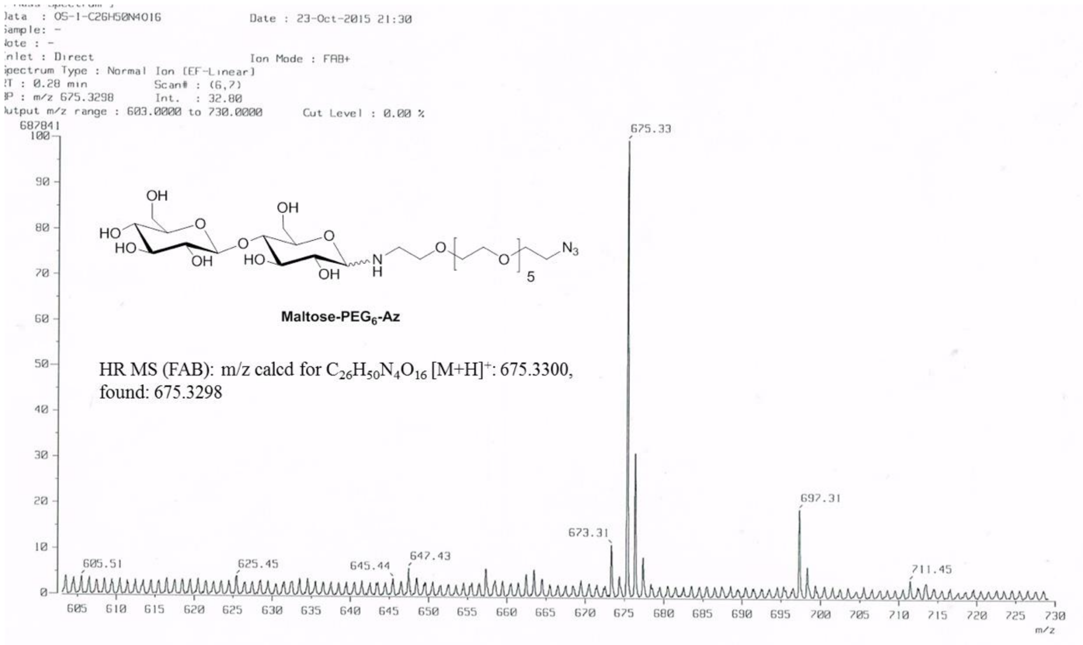

Figure S19. High resolution mass spectroscopic data for maltose-PEG$_6$-azide.

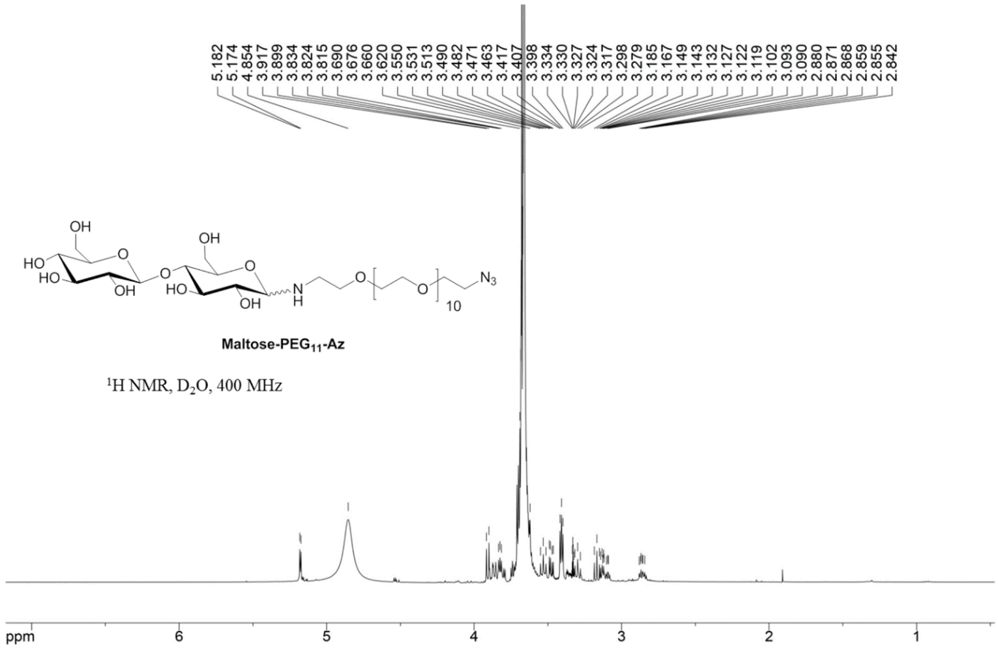

Figure S20. ^{1}H NMR spectra for maltose-PEG$_{11}$-azide.

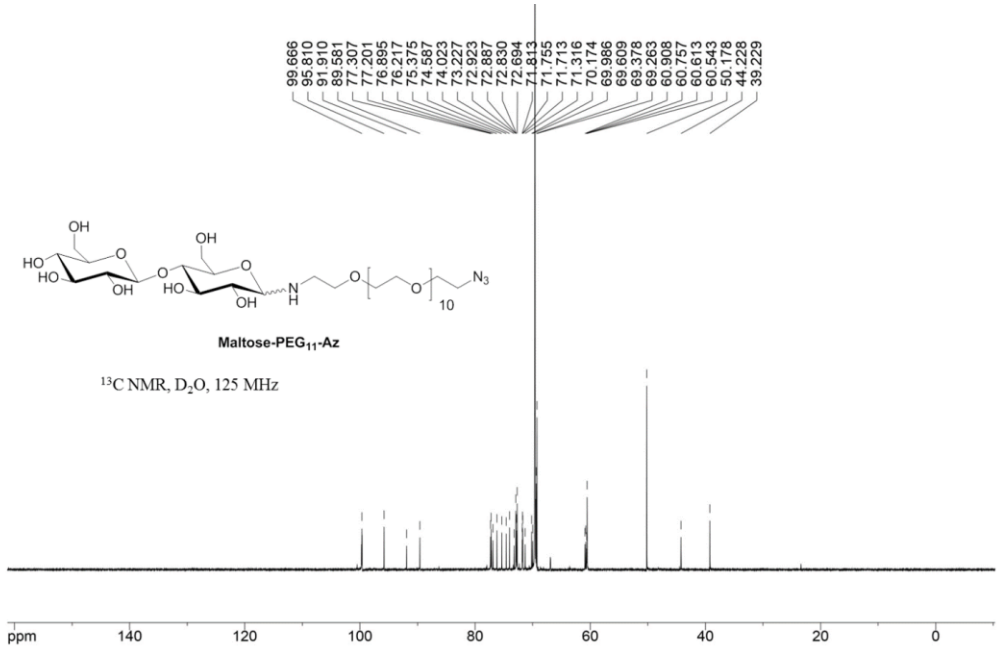

Figure S21. ^{13}C NMR spectra for maltose-PEG$_{11}$-azide.

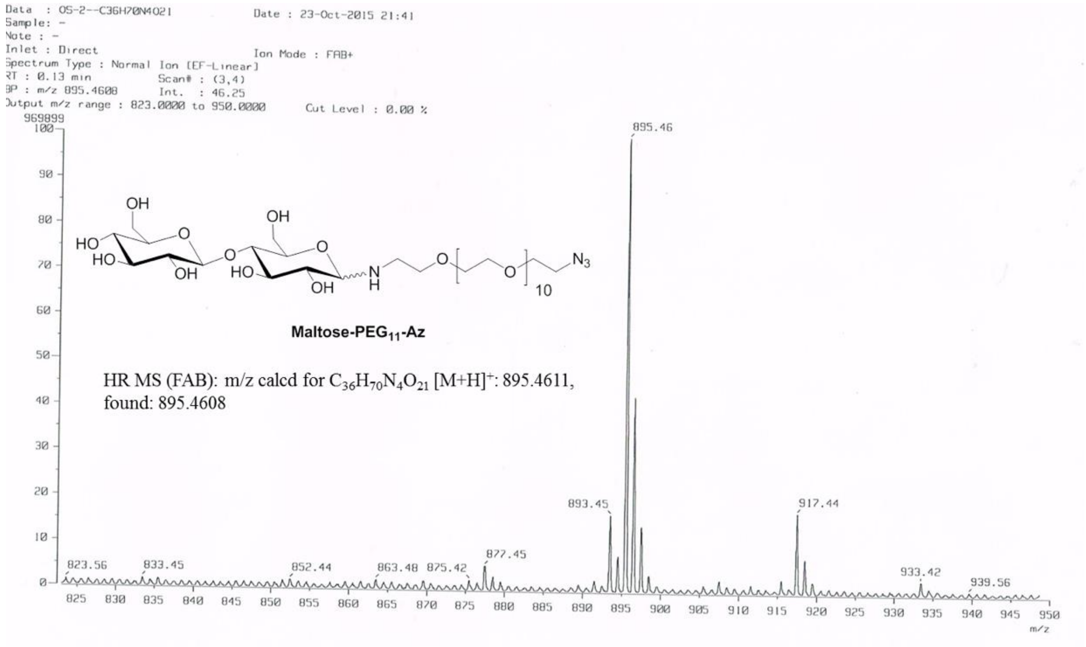

Figure S22. High resolution mass spectroscopic data for maltose-PEG11-azide.

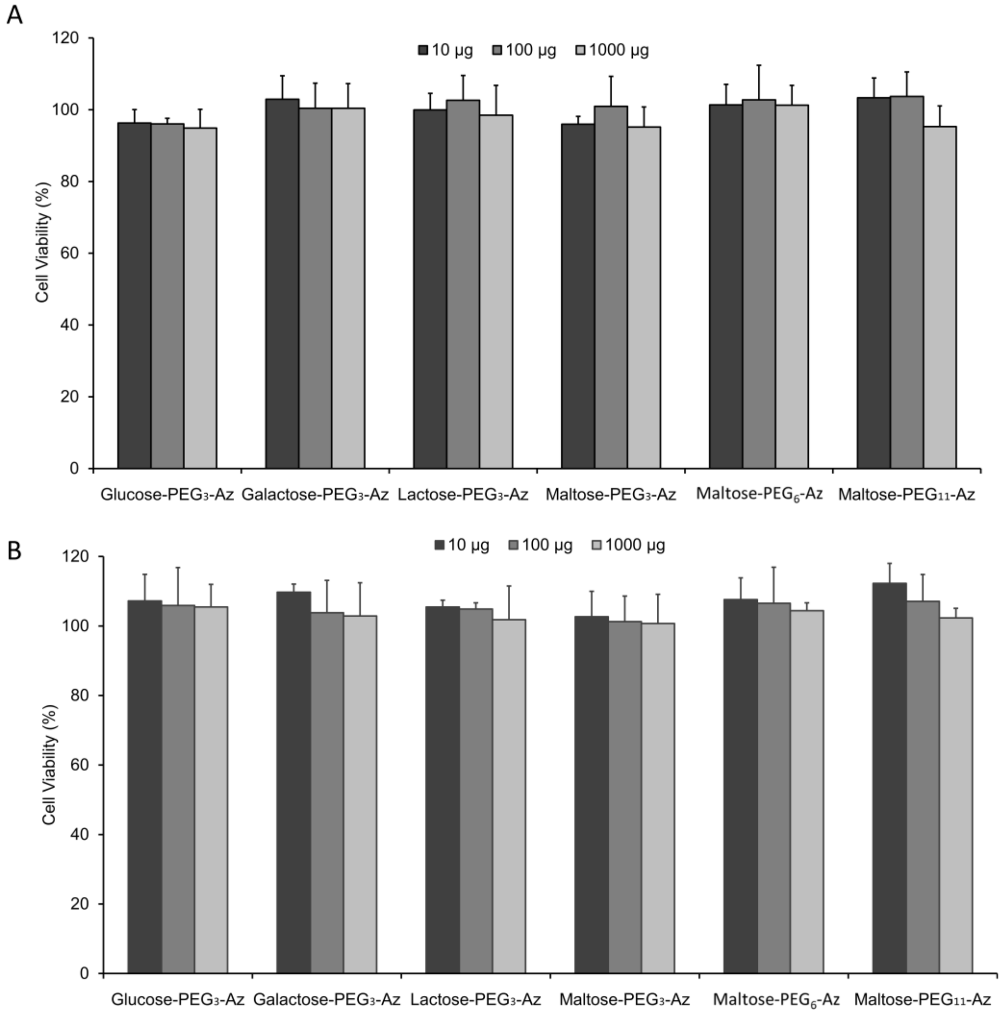

Figure S23. Cell viability of N-glycosyl azides performed in NIH3T6.7 cells by MTT assay; 7 h of treatment (**A**) and 24 h of treatment (**B**).

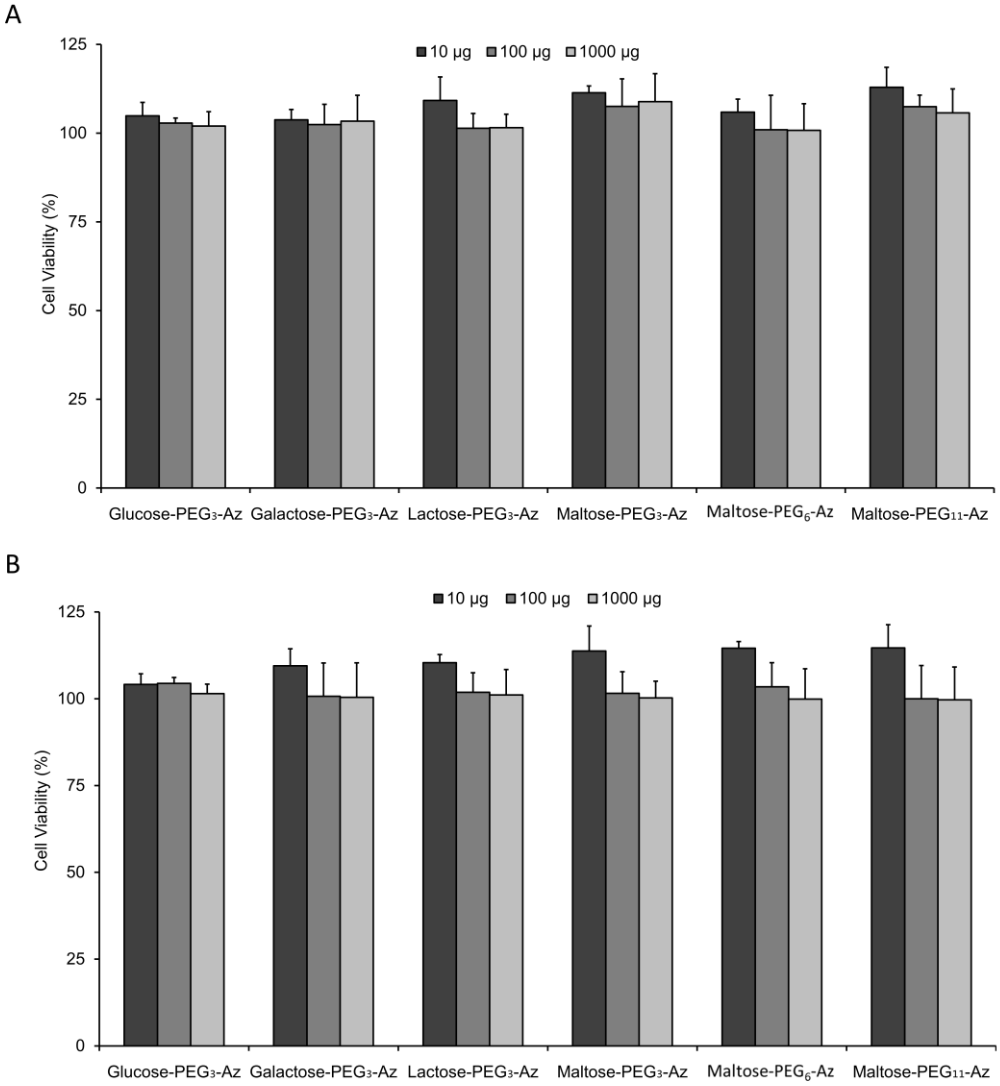

Figure S24. Cell viability of N-glycosyl azides performed in HEK293 cells by MTT assay; 7 h of treatment (**A**) and 24 h of treatment (**B**).

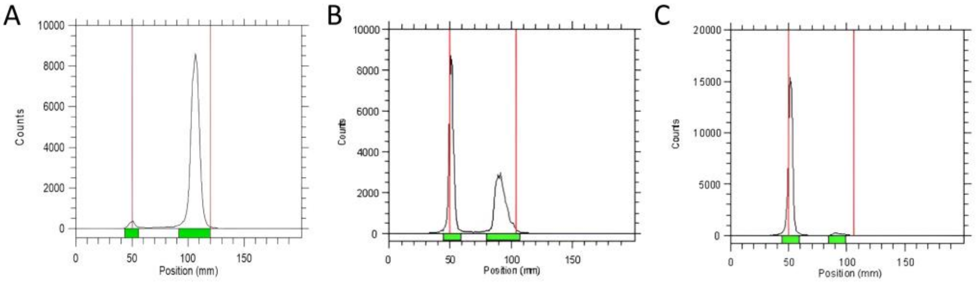

Figure S25. Representative radio thin-layer chromatograms of the radioiodinated tracer DPTA (**5**) (**A**), bioconjugation of radiotracer DPTA with trastuzumab (**B**), and the purified [^{131}I]DPTA-trastuzumab (**6**) (**C**). Radio-TLC analyses were performed on silica with methanol as mobile phase.

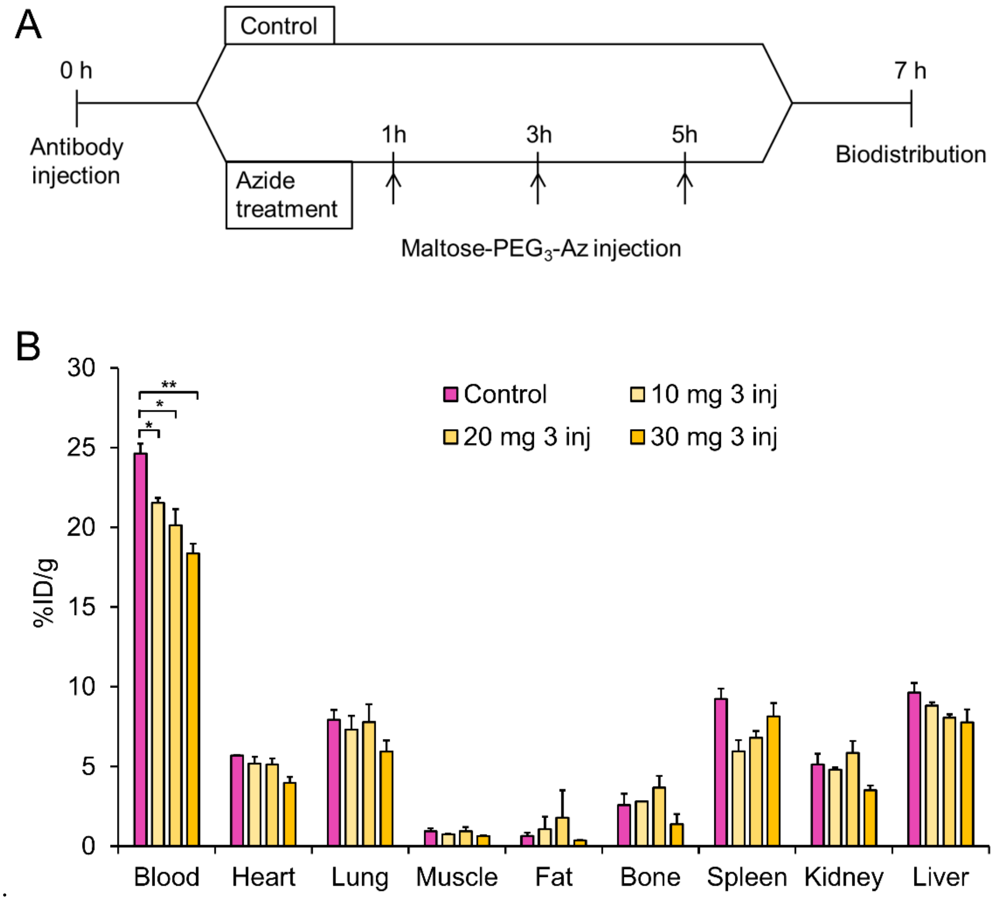

Figure S26. Schematic representation demonstrating study plan (**A**). Biodistribution data of ICR mice after injection of [^{131}I]DPTA-trastuzumab followed by varying the quantity of maltose-PEG3-azide (**B**) (n = 2). *$p < 0.05$ and **$p < 0.001$.

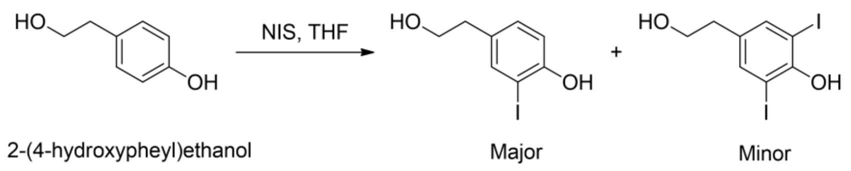

Figure S27. Synthetic scheme of non-radioactive 4-(2-hydroxyethyl)-2-iodophenol.

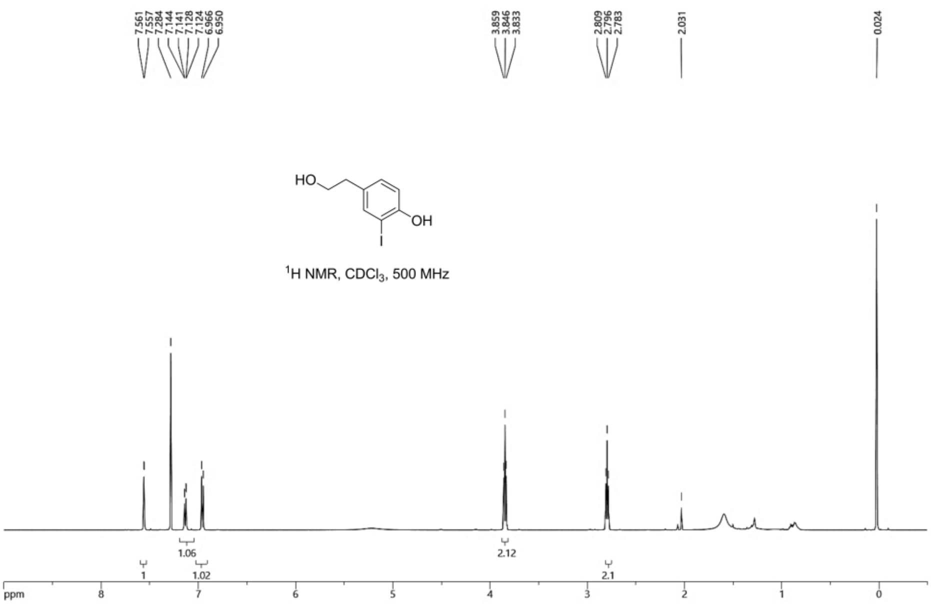

Figure S28. ^{1}H NMR spectrum of 4-(2-hydroxyethyl)-2-iodophenol.

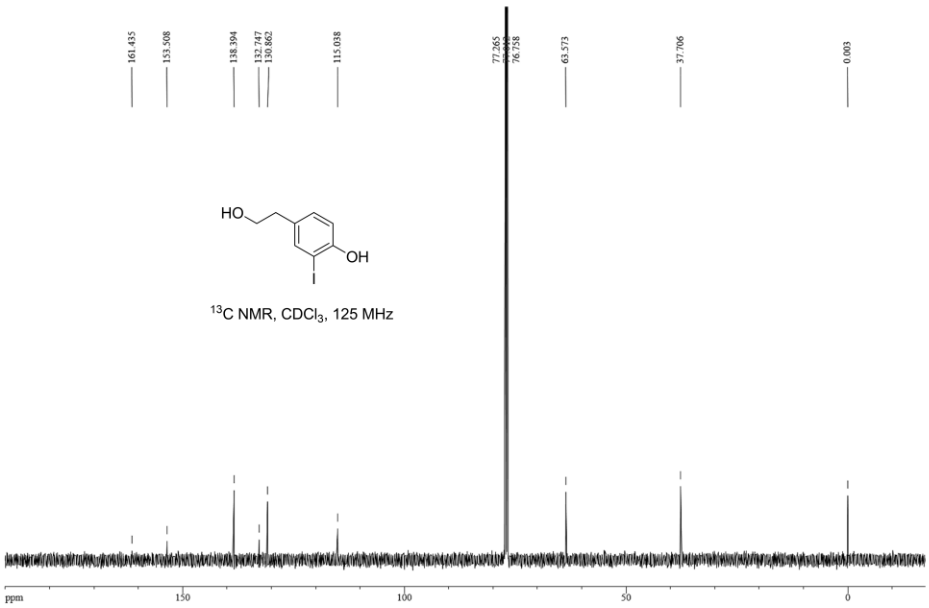

Figure S29. ^{13}C NMR spectrum of 4-(2-hydroxyethyl)-2-iodophenol.

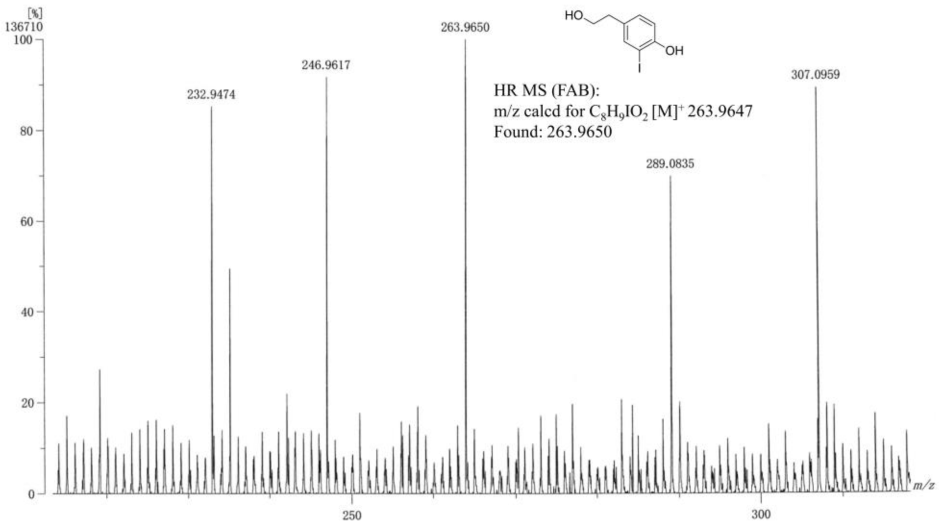

Figure S30. High resolution mass spectroscopic data for 4-(2-hydroxyethyl)-2-iodophenol.

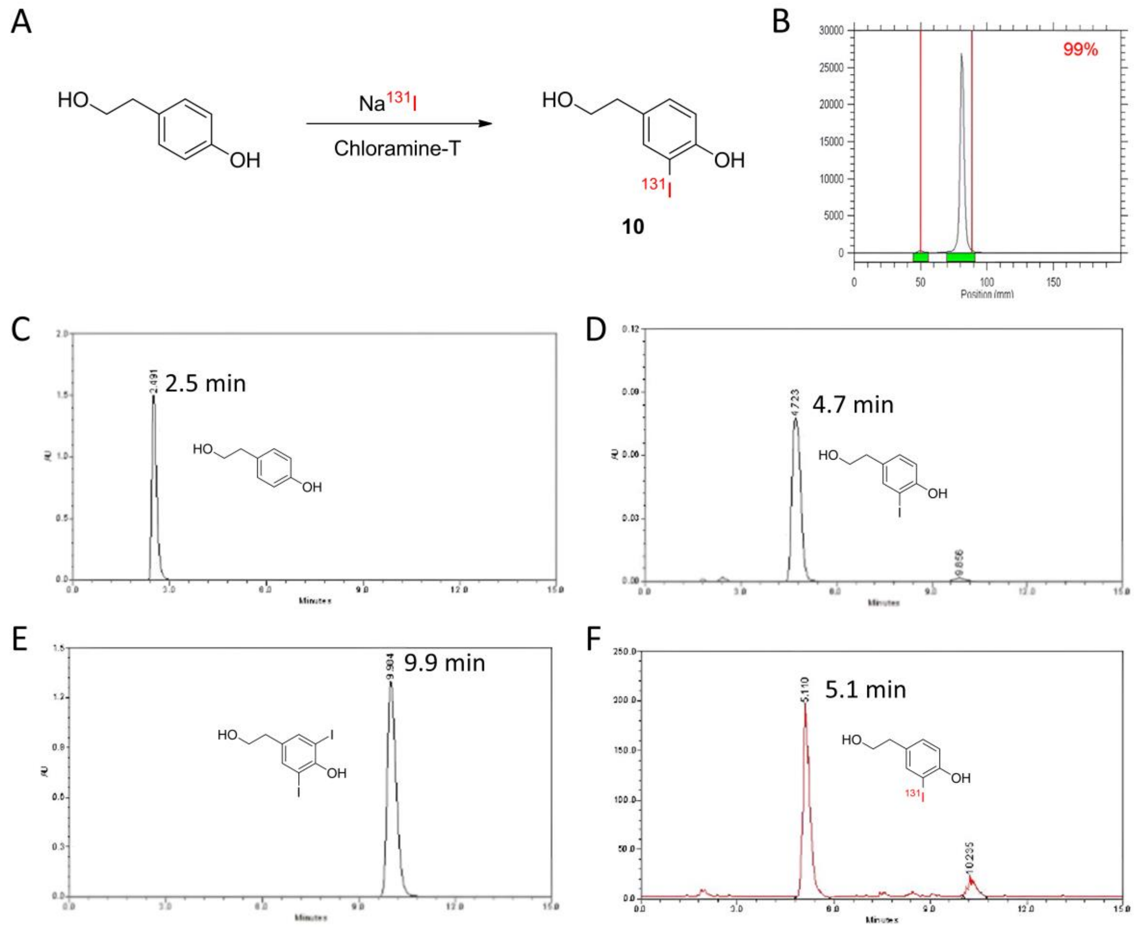

Figure S31. Schematic of radiolabeling of 4-(2-hydroxyethyl)-2-[^{131}I]iodophenol (**10**) (**A**). Radio thin-layer chromatogram of compound **10** performed in silica, ethyl acetate:methanol (95:5) (**B**). UV-HPLC chromatograms of 2-(4-hydroxyphenol) ethanol (**C**), 4-(2-hydroxyethyl)-2-iodophenol (**D**), 4-(2-hydroxyethyl)-2,6-diiodophenol (**E**) and radio-HPLC chromatogram of 4-(2-hydroxyethyl)-2-[^{131}I]iodophenol (**10**) (**F**).

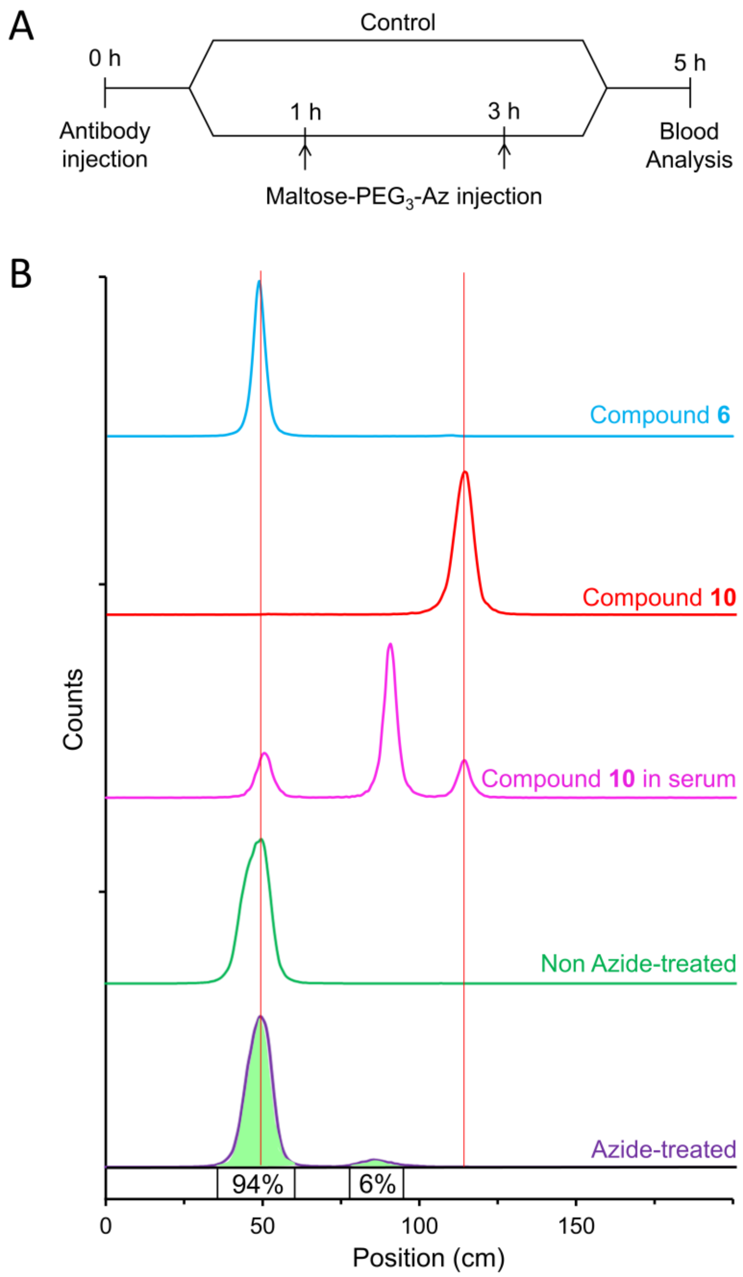

Figure S32. Radio-TLC analysis of in vivo Staudinger ligation in serum of control and azide-treated mice. Purified [^{131}I]DPTA-trastuzumab (**6**) (blue line), 4-(2-hydroxyethyl)-2-[^{131}I]iodophenol (**10**) (red line), non-azide-treated control serum (green line), maltose-PEG$_3$-azide treated serum mixed with 4-(2-hydroxyethyl)-2-[^{131}I]iodophenol (**10**) (pink line), maltose-PEG$_3$-Az treated serum (violet line). All radio-TLC were developed using iTLC and saline.

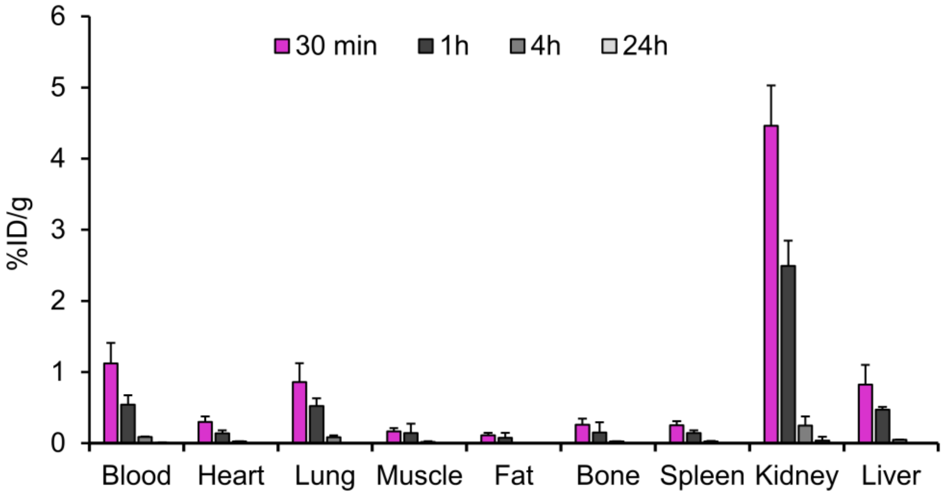

Figure S33. Biodistribution of 4-(2-hydroxyethyl)-2-[^{131}I]iodophenol (**10**) in normal ICR mice (n = 3).
